# Comparative Transcriptome Analysis of Salt Stress-Induced Leaf Senescence in *Medicago truncatula*

**DOI:** 10.3389/fpls.2021.666660

**Published:** 2021-07-09

**Authors:** Shuwei Dong, Lijun Sang, Hongli Xie, Maofeng Chai, Zeng-Yu Wang

**Affiliations:** Grassland Agri-Husbandry Research Center, College of Grassland Science, Qingdao Agricultural University, Qingdao, China

**Keywords:** salt stress, leaf senescence, *Medicago truncatula*, comparative transcriptome analysis, RNA-Seq

## Abstract

Leaves are the most critical portion of forage crops such as alfalfa (*Medicago sativa*). Leaf senescence caused by environmental stresses significantly impacts the biomass and quality of forages. To understand the molecular mechanisms and identify the key regulator of the salt stress-induced leaf senescence process, we conducted a simple and effective salt stress-induced leaf senescence assay in *Medicago truncatula*, which was followed by RNA-Seq analysis coupled with physiological and biochemical characterization. By comparing the observed expression data with that derived from dark-induced leaf senescence at different time points, we identified 3,001, 3,787, and 4,419 senescence-associated genes (SAGs) for salt stress-induced leaf senescence on day 2, 4, and 6, respectively. There were 1546 SAGs shared by dark and salt stress treatment across the three time points. Gene Ontology (GO) and Kyoto Encyclopedia of Genes and Genomes (KEGG) pathway enrichment analyses showed that the 1546 SAGs were mainly related to protein and amino acids metabolism, photosynthesis, chlorophyll metabolism, and hormone signaling during leaf senescence. Strikingly, many different transcription factors (TFs) families out of the 1546 SAGs, including *NAC, bHLH, MYB*, and *ERF*, were associated with salt stress-induced leaf senescence processes. Using the transient expression system in *Nicotiana benthamiana*, we verified that three functional *NAC* TF genes from the 1546 SAGs were related to leaf senescence. These results clarify SAGs under salt stress in *M. truncatula* and provide new insights and additional genetic resources for further forage crop breeding.

## Introduction

In nature, leaf senescence is an inevitable process by which plants reallocate nutrients, thus altering their metabolism and energy cycles to redirect more nutrients from senescent or dying leaves to new leaves, flower development, seed maturation, or other tissues and storage organs (Hörtensteiner and Feller, 2002; Yoshimoto et al., 2009; Bar-Dror et al., 2011). Therefore, leaf senescence is a key developmental process that ensures optimal plant performance and directly enhances plant adaptability and viability in the face of stress (Peleg and Blumwald, 2011). However, premature senescence caused by stress can significantly result in yield loss and food quality reduction (Uauy et al., 2006; Yamada et al., 2009; Gregersen et al., 2013). Through delaying the senescence of functional leaves by 1 day, it has been calculated that yields can be increased by 2–10% in major crops such as maize, soybeans, cotton, rice, and wheat (Buchanan-Wollaston et al., 2003; Liu et al., 2015; Schippers et al., 2015). As for forage crops such as alfalfa, most of the nutrients are indeed stored in the leaves. Therefore, leaf senescence has a more direct impact on the production of high-quality forage (Du et al., 2020). As a result, many researchers focused on improving crop yields and agricultural productivity by delaying the leaf senescence process (Breeze, 2020; Bian et al., 2021; Li et al., 2021).

Leaf senescence is a highly complex process that is tightly regulated at multiple levels, including the chromatin level, the mRNA level (via transcriptional and posttranscriptional regulation), and the protein level (via translational and posttranslational regulation). It has been noted that along with leaf senescence, thousands of leaf senescence-associated genes (SAGs) such as transcription factors (TFs) are induced (Buchanan-Wollaston, 1997; Woo et al., 2001). A wide range of TF families (e.g., NAC, WRKY, CDF, bZIP, and MYB) has been shown to participate, often critically, in the regulation of senescence in plants (Zhou et al., 2011b; Rushton et al., 2012; Jaradat et al., 2013; Kim et al., 2013; Mao et al., 2017; Woo et al., 2019; Xu et al., 2020). These regulatory networks are interconnected with multiple crosstalk pathways, thus enabling responses to and regulation on various internal and external environmental senescence signals (Buchanan-Wollaston et al., 2005; Lim et al., 2007; Park et al., 2007; Breeze et al., 2011; Allu et al., 2014). Unfavorable environmental conditions include prolonged drought, darkness, salinity, heat, cold, pests, and diseases (Wingler et al., 2006; Lim et al., 2007; Parlitz et al., 2011; He et al., 2018). Salt stress is one of the common abiotic stresses limiting plant growth and development, and it has become a severe problem restricting crop production as well as ecological environment construction (Lutts et al., 1996; Munné-Bosch and Alegre, 2004). Salt stress can induce senescence in plant leaves, but the regulatory mechanisms are not fully understood (Munns and Tester, 2008; Balazadeh et al., 2010; Allu et al., 2014).

Over the last decade, significant progress has been made in understanding the key molecular principles of leaf senescence in model plants including *Arabidopsis thaliana* (Lim et al., 2003; Lin and Wu, 2004; Buchanan-Wollaston et al., 2005; Breeze et al., 2011), rice (Lee et al., 2001), tobacco (Pageau et al., 2006) and other crops, such as wheat (Uauy et al., 2006), corn (Zhang et al., 2014), and cotton (Kong et al., 2013). Few such studies have been performed in leguminous forage species. *Medicago truncatula*, a close relative of alfalfa (*Medicago sativa*), has been developed as an excellent model legume system, especially for studying forage crop improvements. To elucidate salt-induced leaf senescence in a time-saving and well-targeted way, salt stress-induced detached leaf senescence assays have been applied as useful tools for studying senescence in *Arabidopsis* and rice (Sakuraba et al., 2018).

To discover the key regulators of leaf senescence in forage crops, in this study, we examined salt- and dark-induced leaf senescence in *Medicago truncatula* through collecting and analyzing physiological, biochemical, and transcriptional data over the time course of leaf senescence. This work presents detailed expression profiles of leaf senescence induced by salt stress in *Medicago truncatula*. Many key active regulatory networks and senescence signaling pathways were illustrated. Several important TFs involved in this process were also characterized using the transient expression system in *Nicotiana benthamiana*. Our work reveals new molecular regulatory mechanisms of leaf senescence in *Medicago truncatula* and provides a valuable genetic resource to improve the biomass and quality of forage crops under salt stress.

## Materials and Methods

### Plant Material and Stress Treatments

*Medicago truncatula* ecotype R108 was used as the experimental plant material. For seed germination, we used sandpaper to lightly scratch the coats of *Medicago truncatula* seeds that had already been vernalized for 2 days. Seeds were then transferred to dishes with moistened filter paper and incubated in a light incubator for 7 days before being transferred to hydroponic growth cultivation in Hoagland's nutrient solution; during this period the culture medium was changed every 3 days. Plants were placed in an artificial climate incubator with a 16 h photoperiod, day/night temperatures of 25°C/22C, and a relative humidity of 60–70%.

Leaves were sampled when plants were 34 days old, and the sampling site was the third compound leaf of the *Medicago truncatula* plant (Supplementary Figure 1). The removed leaves were immediately placed in Petri dishes containing different treatment solutions. The Petri dishes were then placed in light or dark conditions according to the experimental design, with the same growth conditions as those generally used for the hydroponic growth of plants. The salt stress treatment fluid was the same as that used for the control treatments (2.4 g of Murashige–Skoog medium, 3 mM MES buffer, pH 5.8), except for its 100, 150, or 200 mM NaCl concentrations achieved by addition of NaCl.

We collected all samples on day 0, 2, 4, and 6 and stored them at −80°C after quickly freezing them in liquid nitrogen. Sampled materials were used to assay physiological indicators and for transcriptomic sequencing.

### Physiological Measurements

To determine the chlorophyll content, 0.1 g samples of previously frozen leaves were weighed, frozen, ground, and placed in 96% (*v*/*v*) acetone. The 3-h extraction was performed at room temperature under dark conditions, and then, samples were centrifuged at 4°C 6,000 rpm for 30 s, then the supernatant was collected. The chlorophyll content in the supernatant was quantified spectrophotometrically (Zhang and Guo, 2018).

The H_2_O_2_ contents were determined spectrophotometrically, while the ABA content was determined by high performance liquid chromatography.

At least three biological replicates were analyzed for each of the above trials. All data were subjected to single-factor ANOVA using SPSS 26 (IBM Corp., Armonk, NY, USA). The differences among the different treatment means were evaluated using Duncan's multiple range test, and *P* < 0.05 were considered statistically significant. All charts were created with Excel 2019 (Microsoft Corp., Redmond, WA, USA).

### RNA Isolation, Quantification, and Quality Controls

Total RNA was extracted from the tissue using TRIzol® Reagent (Plant RNA Purification Reagent for plant tissue; Invitrogen Corp., Carlsbad, CA, USA) according the manufacturer's instructions and genomic DNA was removed using DNase I (TaKaRa, Kusatsu, Japan). Then, RNA quality was determined with a 2100 Bioanalyser (Agilent; Santa Clara, CA, USA) and quantified using the ND-2000 platform (NanoDrop Technologies, Wilmington, DE, USA). Only high-quality RNA samples [OD_260/280_ = 1.8–2.2, OD_260/230_ ≥ 2.0, RNA integrity number (RIN) ≥ 6.5, 28S:18S ratio ≥ 1.0, >2 μg samples] were used to construct sequencing libraries.

### cDNA Library Construction and RNA Sequencing

RNA-Seq transcriptome libraries were prepared using the TruSeq^TM^ RNA sample preparation Kit from Illumina (San Diego, CA, USA) with 1 μg of total RNA. In brief, messenger RNA was first isolated using the polyA selection method with oligo(dT) beads and then fragmented with fragmentation buffer. Second, double-stranded cDNA was synthesized using a SuperScript double-stranded cDNA synthesis kit (Invitrogen) with random hexamer primers (Illumina). Then, the synthesized cDNAs were subjected to end-repair, phosphorylation, and “A” base addition according to Illumina's library construction protocol. Libraries were size selected for 200–300 bp cDNA target fragments using 2% Low Range Ultra Agarose electrophoresis followed by PCR amplified using Phusion DNA polymerase (New England Biolabs, Ipswich, MA, USA) for 15 cycles. After quantification with a TBS380 fluorometer (Turner BioSystems, Sunnyvale, CA, USA), the paired-end RNA-seq sequencing library was sequenced with the Illumina HiSeq xten/NovaSeq 6000 sequencer (2 × 150 bp read length).

The raw paired end reads were trimmed and quality-filtered by Fastp version: 0.19.5 (https://github.com/OpenGene/fastp) for acquiring clean reads.

All the obtained high-quality and clean reads were mapped against the reference genome of *Medicago_truncatula* (reference genome version is MedtrA17_4.0; Reference genome source can be reached via http://plants.ensembl.org/Medicago_truncatula/Info/Index) by Mapping tools hisat2 (Version 2.1.0, http://ccb.jhu.edu/software/hisat2/index.shtml), and the matching rate ranged from 6.44 to 90.06%.

To evaluate the similarity of RNA-Seq data from different samples, the mapped RNA-Seq data from different samples were first subjected to a standardized analysis (Z-score) and then to a principal component analysis (PCA) with the analysis software Python.

All information related to project sample information, raw data, quality control data, and comparison data can be found in Supplementary Table 1. Reference genome annotation information are listed in Supplementary Table 2.

### Differential Gene Expression Analysis and Functional Enrichment

To identify differentially expressed genes (DEGs) between two different samples, the expression level of each transcript was calculated according to the transcripts per million reads (TPM) method. The expression quantification software tool RSEM (RNA-Seq by Expectation-Maximization, http://deweylab.github.io/RSEM/) was applied to quantify gene abundances for each group and each time point, respectively, using the average transcripts per million reads (TPM) values of biological replicates as a quantitative metric.

R statistical package software EdgeR (Empirical analysis of Digital Gene Expression in R, (http://www.bioconductor.org/packages/2.12/bioc/html/edgeR.html) was adapted to perform differential expression analysis. The threshold values for screening DEGs were |log2FC| ≥ 1 or ≤ −1 & *P-adjust* < 0.05. The method of multiple test correction is BH (FDR correction with Benjamini and Hochberg). In order to control the probability or frequency of error in the overall inference result, the *p*-value obtained from the statistical test is corrected, i.e., multiple test correction is performed, and the corrected *p*-value is called P-adjust.

In addition, Enrichment analysis using Fisher's precision test, functional-enrichment analysis including GO and KEGG enrichment analyses, were performed to identify which DEGs were significantly enriched within specific GO terms and metabolic pathways at a BH-corrected *P*-value (*p*-adjust) < 0.05 threshold compared with the whole-transcriptome background. GO functional enrichment and KEGG pathway analysis were conducted using Goatools (https://github.com/tanghaibao/Goatools) and KOBAS (http://kobas.cbi.pku.edu.cn/home.do), respectively. The GO and KEGG enrichment analysis of senescence-associated genes (SAGs) is detailed in Supplementary Tables 3, 4, respectively. Specific information on the software names, version details, and URL links used in this study are shown in Supplementary Table 5.

### Quantitative Real-Time PCR Analysis

In order to verify the expression patterns of genes observed in the RNA-seq analysis, we used the total RNA of all samples subjected to RNA-Seq analysis for qRT-PCR. Sixteen DEGs were selected for qRT-PCR detection, and the *MtACTIN* gene was used as a reference gene. RNAs were reverse transcribed into cDNAs using M5 Super Plus qPCR RT kit with gRNA remover (mei5 Biotech Co., Ltd; Beijing, China). ChamQ SYBR color qPCR Master Mix (Vazyme Biotech Co., Ltd; Nanjing, China) was used for qRT-PCR, and three technical repeats were performed for each reaction. All primers were designed using Primer 5.0 software and are listed in Supplementary Table 6.

### Functional Verification of Genes by *Agrobacterium*-Mediated Transient Expression

We selected 9 genes for transient expression in tobacco (*Nicotiana benthamiana*), among which *SGR* was selected as a known positive control. Using the cDNAs from the cDNA library as templates, nine target genes were cloned and sequenced to verify the sequence accuracy. Then, using seamless DNA cloning (ClonExpress® Ultra One Step Cloning Kit, Vazyme Biotech Co., Ltd), the target genes were ligated to the pBI121 vector. The ligation products were transformed into *E*. *coli* strain DH5α (Vazyme Biotech Co., Ltd), and a positive *E*. *coli* colony was selected and verified by sequencing. The plasmid was extracted and then transformed into *Agrobacterium tumefaciens*, and a single positive *Agrobacterium tumefaciens* colony was chosen for *Nicotiana benthamiana* injection using the method described by Li et al. (2008).

The software tool CE Design (http://www.vazyme.com) was used to design specific amplification primers (Supplementary Table 6).

### Transcription Factor Family Analysis

TFs usually consist of a DNA-binding domain (DBD), a transcription-activating domain (TAD), and other TF-binding domains (e.g., signal sensing domain). Depending on their functional domains, TFs can be grouped into different families. TFs in the same family generally contain the same DBD and different TADs. Analysis of the domain information contained in gene transcription products was used for TF prediction and family analysis of genes. We used Plant TFDB 4.0 (http://planttfdb.cbi.pku.edu.cn/) for TF analysis. For blastp, an *E* < 1e-5 threshold was used. For a detailed table of TF statistics, please see Supplementary Table 7. We used the software FIMO to predict the target genes of TF MTR_3g088110 based on the information of transcription factor binding sites included in the Plant TFDB database. We screened target genes with high correlation (correlation> 0.5, *P* < 0.05) with TF MTR_3g088110 from 3001,3787,4419 SAGs (**Figures 4A–C**). The information related to the target genes obtained at 2, 4, and 6 days of prediction is shown Supplementary Table 8.

### Short Time-Series Expression Miner and Functional Similarity Analyses

Time-series analysis (time sequence expression analysis) involves the comparison of gene expression on the same (or similar) samples at different time points, in order to observe changes in gene expression between each time point, and to elucidate the patterns of interdependence between genes. Short Time-series Expression Miner (STEM, Version 1.3.11) with a *p* < 0.05 threshold was used for time-series expression trend analysis (Supplementary Table 9).

### Microarray Significant Profiles Analysis

The maSigPro was applied to perform the analysis of gene expression data at different time point. MaSigPro (Version 1.56.0, http://www.bioconductor.org/packages/release/bioc/html/maSigPro.html) is an R package for analyzing time series data based on multiple linear regression models to fit the time, experimental conditions, and gene expression, then apply a stepwise regression method to find the best combination of independent variables. Time-series difference analysis based on maSigPro was used to obtain the genes that have changed differentially throughout the sample of time series nodes (Supplementary Table 10).

## Results

### Physiological and Biochemical Changes During Salt-Induced Leaf Senescence

To illustrate the effects of salt stress on leaf senescence, we collected fully expanded single leaves without senescence signs from 5-week-old *Medicago truncatula* plants (Supplementary Figure 1A) as experimental materials. Different concentrations of salt (NaCl) treatments were investigated in this experiment. As the salt concentration increased, the leaf senescence developed more dramatically (Supplementary Figure 1B). Based on our preliminary experimental results, the 150 mM NaCl treatment was observed to induce leaf senescence similar to that observed under the dark treatment. Dark-induced leaf senescence has been reported to be similar to age-dependent leaf senescence when plants develop normally (Sobieszczuk-Nowicka et al., 2018).

**Figure 1:**
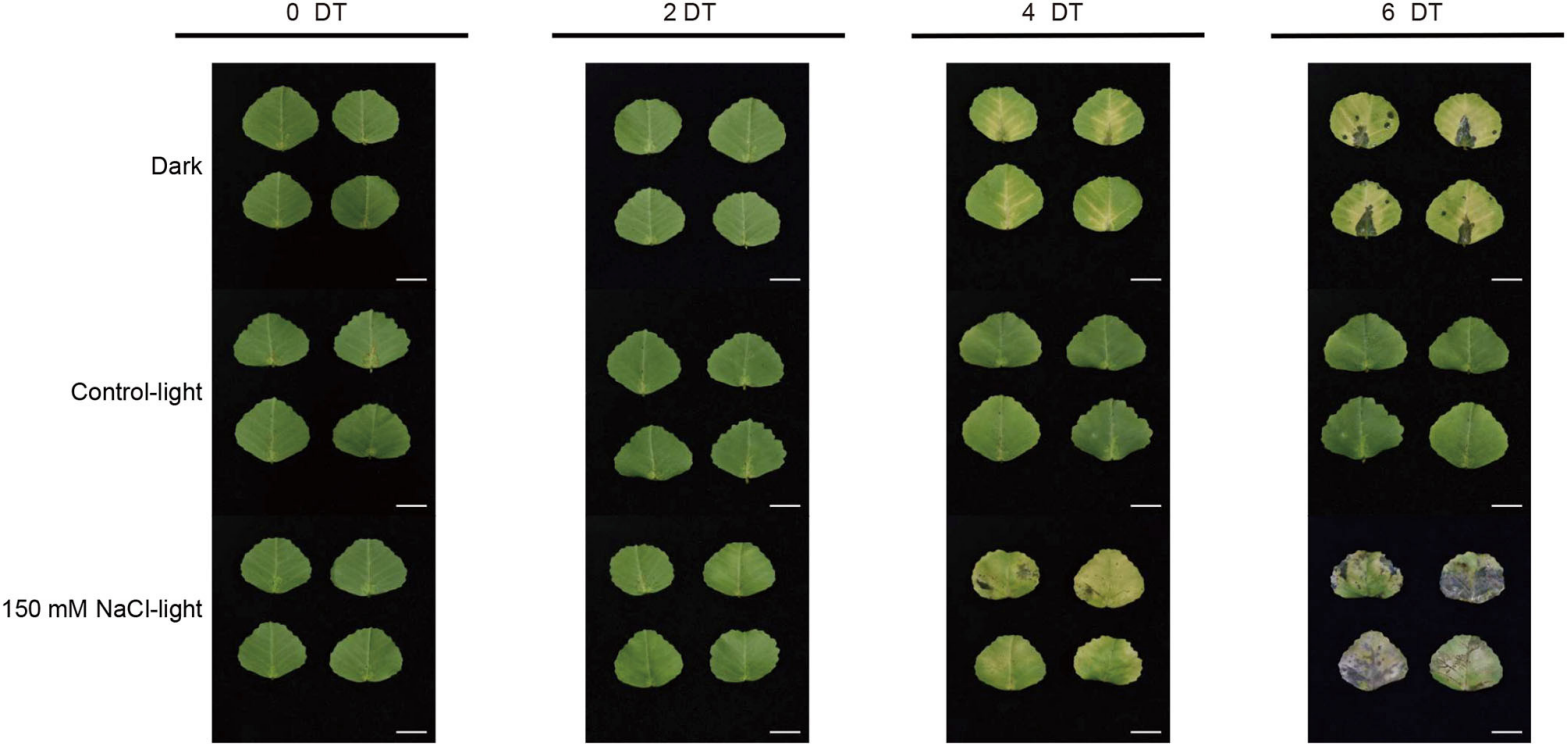
Salt stress-induced leaf senescence in *Medicago truncatula*. The leaf color and senescence progression of detached *M*. *truncatula* leaves under dark (Dark), normal light (Control-light), and 150 mM NaCl (150 mM NaCl-light) conditions for 0, 2, 4, and 6 days. DT, day(s) of treatment. Scale bar, 1 cm.

To remove the background effect of the development process, we established a light control group (control-light). To better screen the senescence-related genes, we set up a dark treatment group as a positive control in the salt-induced senescence experiment performed on isolated leaves. Thus, this experimental design could exclude the negative control conditions and be compared with the positive control senescence conditions. The salt-induced senescence experiments were performed under conditions with 150 mM NaCl and light. It was observed that the detached leaves showed different senescence phenotypes across the different groups (Figure 1). In the salt-induced group, the leaves started to turn yellow on day 4 and gradually turned 50–90% yellow with eroded, partially blackened, or transparent leaves on day 6. In the dark-induced group, leaves showed signs of yellowing on day 4 and increased yellowing on day 6; however, in the light control group, entire leaves stayed green and showed no signs of senescence until day 6.

**Figure 2:**
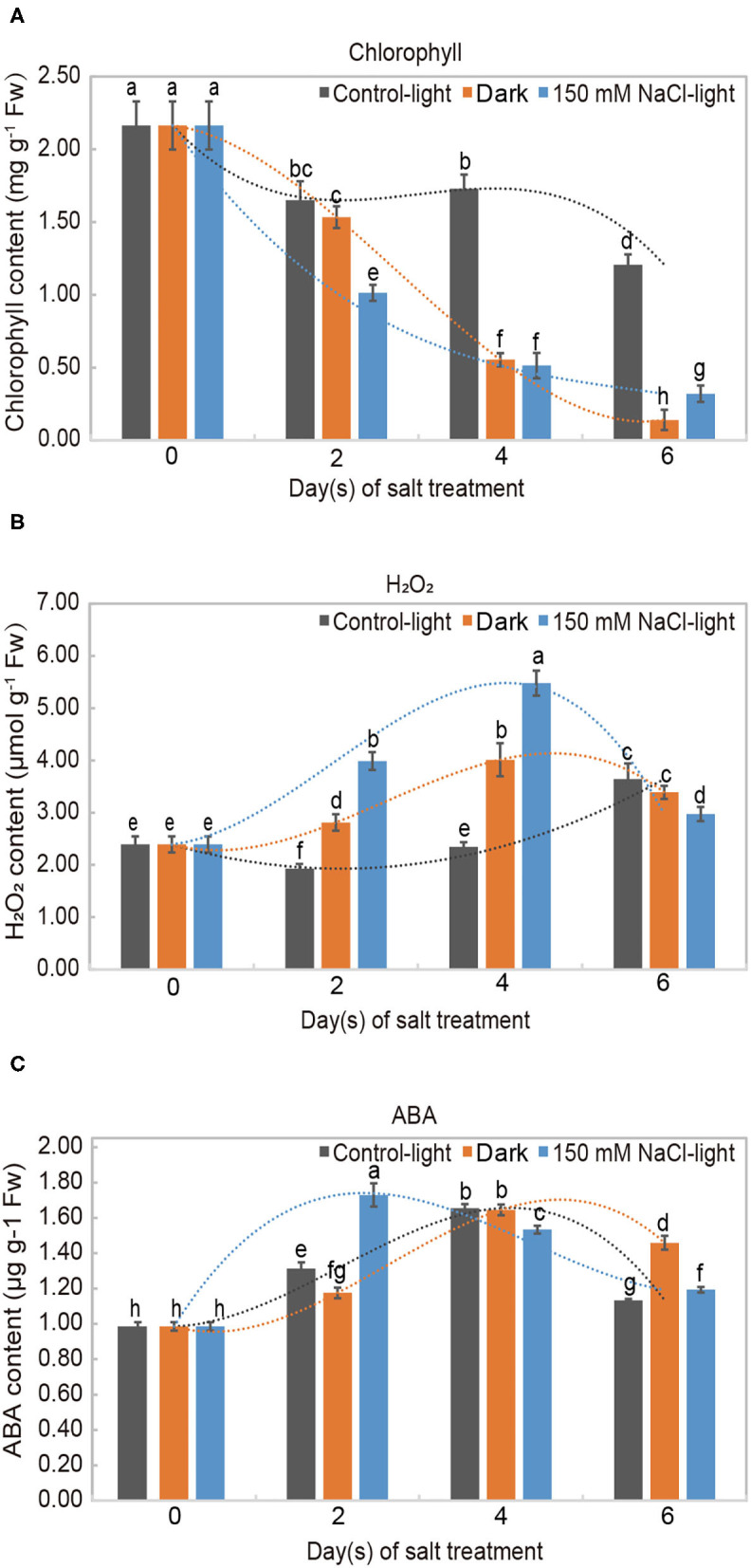
Physiological analysis of salt-induced leaf senescence in *Medicago truncatula*. Levels of **(A)** chlorophyll (a+b), **(B)** hydrogen peroxide (H_2_O_2_), and **(C)** abscisic acid (ABA) in detached *Medicago truncatula* leaves exposed to different conditions during leaf senescence. The values are presented as the mean ± SE values of three independent biological replicates per time point. Different letters indicate significant differences among treatments according to an analysis of variance (ANOVA, *P* < 0.05). Error bars correspond to standard errors. FW indicates fresh weight.

We investigated the physiological and biochemical responses of *in vitro* leaves treated with 150 mM NaCl through measuring chlorophyll, H_2_O_2_, and abscisic acid (ABA) contents. Chlorophyll is commonly used as a marker and representative indicator of plant senescence to evaluate the leaf senescence process. The chlorophyll content decreased significantly in both the salt-induced group and the dark-induced group and was only slightly degraded in the light control group (Figure 2A). H_2_O_2_ is an important biochemical indicator that reflects the emergency response of oxidation under stress. H_2_O_2_ content showed a complex profile throughout leaf senescence. In the salt-induced group, H_2_O_2_ content increased initially up to its maximum on day 4 and then decreased. The levels of H_2_O_2_ in the dark-induced group and salt-induced group followed similar trends (Figure 2B). ABA content increased during leaf senescence in both the salt or dark-induced groups and the control group. In the salt-induced group, ABA content peaked on day 2 and then decreased, but remained above the initial value (Figure 2C).

### Transcriptomic Analysis Identified Thousands of Differentially Expressed Senescence-Associated Genes at Different Time Points

We collected leaves sampled on day 0, 2, 4, and 6 of treatment for sequencing, with three biological replicates for each group at each time point, thus obtaining a total of 222.57 Gb of clean data (Supplementary Table 1) from the 10 groups (30 samples in total) of leaf samples. The clean data for each sample exceeded 5.91 Gb. Approximately 85% of clean reads matched the reference genomic sequence, and the Q30 base percentage was above 91.87%.

**Figure 3:**
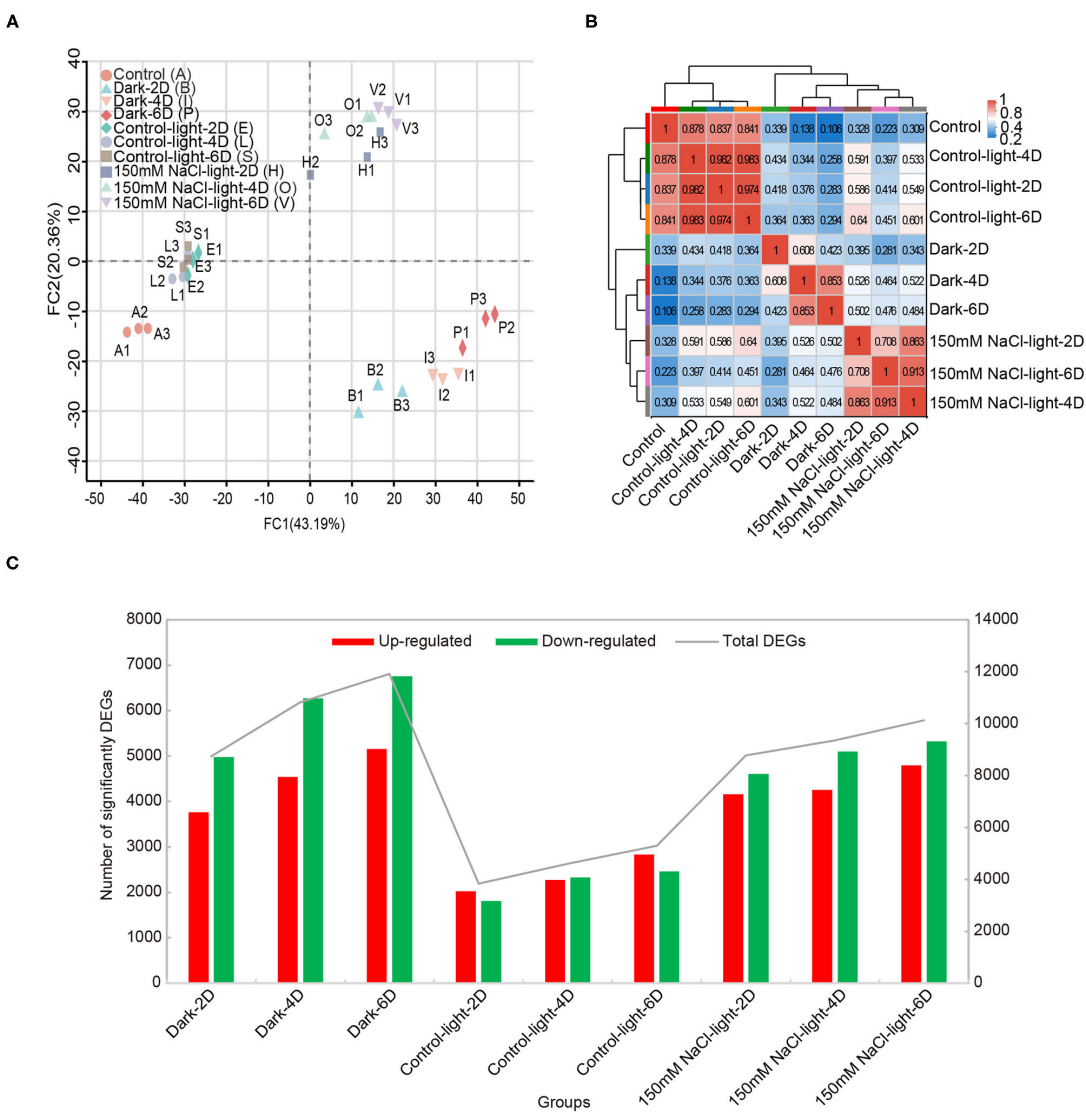
Transcriptomic overview of salt stress-induced detached leaf senescence in *Medicago truncatula*. **(A)** Principal component analysis (PCA) plot of transcriptome profiles from different conditions. **(B)** Correlation analysis showed the direct association between samples in the same treatment. **(C)** The number of differentially expressed genes that were up- or down-regulated during leaf senescence compared with 0 day control prior to treatment, as assessed using the difference analysis software edgeR at thresholds of |log2FC| ≥ 1 or ≤ −1 & *P*-adjust < 0.05.

Principal component analysis (PCA) showed that replicate samples from the same group were tightly clustered together, suggesting that each biological replicate sample had low variability. Among different groups, the transcriptomic data differed substantially across time points, suggesting that the salt stress treatment caused significantly differential expression of genes (Figure 3A). Validation of RNA-seq data by quantitative real-Time PCR (qRT-PCR) analysis revealed similar expression profiles (Supplementary Figure 2). The observed high inter-sample correlation also validated the experimental design (Figure 3B). Using the software edgeR with screening thresholds of |log2FC| ≥ 1 or ≤ −1 together with *P* < 0.05, the differentially expressed genes (including up- and down-regulated genes) obtained from each group compared to the control group were analyzed (Figure 3C, Supplementary Table 11).Compared with the 0 day time point, both salt- and dark-induced groups had large amounts of up- and down-regulated genes, ranging from 3,700 to 6,700, while in the light control group differentially expressed genes numbered ~2,000 for both up- and down-regulated genes (Figure 3C).

**Figure 4:**
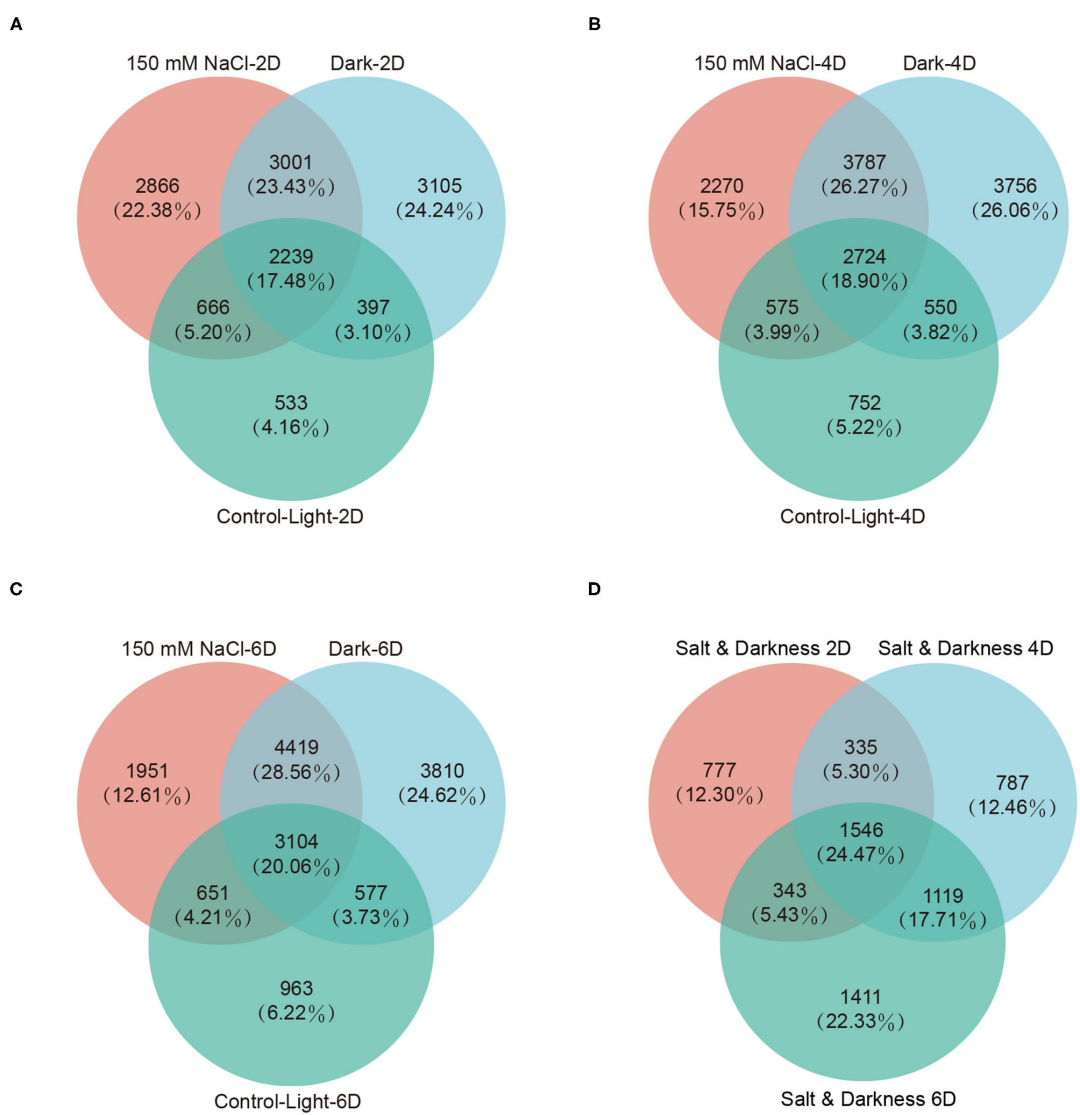
Differentially expressed senescence-associated genes (SAGs) analysis. Differentially expressed SAGs in detached leaves under darkness and 150 mM NaCl treatment on day 2 **(A)**, 4 **(B)**, and 6 **(C)**. **(D)** Venn diagram of SAGs shared by the darkness and 150 mM NaCl treatments on day 2 (**A**, 3001), 4 (**B**, 3787), and 6 (**C**, 4419). There were 1546 SAGs shared by darkness and 150 mM NaCl treatments across three time points.

To obtain genes associated with leaf senescence, we generated Venn diagrams of the differentially expressed genes at each time point under different treatments to identify the genes that were induced in both the salt- and dark-induced groups but were not identified in the light control group. This analysis identified 3,001, 3,787, and 4,419 senescence-associated genes (SAGs) on day 2, 4, and 6, respectively (Figures 4A–C, Supplementary Table 12). Then, we assessed the Venn diagrams of the differentially expressed SAGs shared by salt- and dark-induced groups at three time points, thus obtaining 1546 SAGs shared by the three time points (Figure 4D).

### Gene Ontology Enrichment and Kyoto Encyclopedia of Genes and Genomes Pathway Enrichment Analysis of SAGs Throughout Leaf Senescence

We investigated 1546 differentially expressed SAGs by using their GO classification. According to the GO enrichment analysis results, differentially expressed genes were mainly enriched within the cellular carbohydrate biosynthetic process, plastid membrane organization, sulfur compound biosynthetic process, and small molecule catabolic process categories (Table 1). The directed acyclic plots of GO terms revealed that senescing leaves subjected to salt stress *in vitro* were significantly affected in terms of the genes associated with the structure and function of intracellular chloroplasts and thylakoids, resulting in a dramatic decrease in leaf photosynthesis (Supplementary Figure 3).

**Table 1:** Gene ontology enrichment and Kyoto encyclopedia of genes and genomes pathway enrichment analysis of SAGs (Figure 4D, 1546 genes) shared by darkness and salt-induced leaf senescence at three time points.

**No**.	**GO Id**	**GO term**	**Sample gene number**			**Background gene number**	**Rich factor**	***P*-value**	***P*-adjust**
			**Total**	**Up**	**Down**				
1	GO:0009658	BP-chloroplast organization	23	3	20	149	0.154362416	2.85437E-07	0.000229793
2	GO:0016054	BP-organic acid catabolic process	21	16	5	150	0.14	3.17216E-07	0.000229793
3	GO:0046395	BP-carboxylic acid catabolic process	21	16	5	150	0.14	3.17216E-07	0.000229793
4	GO:0042793	BP-plastid transcription	14	0	14	40	0.35	3.52359E-07	0.000229793
5	GO:0000023	BP-maltose metabolic process	13	0	13	50	0.26	3.73532E-07	0.000229793
6	GO:0044272	BP-sulfur compound biosynthetic process	22	6	17	182	0.126373626	3.81082E-07	0.000229793
7	GO:0009668	BP-plastid membrane organization	34	0	34	110	0.309090909	4.03439E-07	0.000229793
8	GO:0061024	BP-membrane organization	34	0	34	248	0.137096774	4.22901E-07	0.000229793
9	GO:0034637	BP-cellular carbohydrate biosynthetic process	34	0	26	322	0.093167702	5.68159E-07	0.000229793
10	GO:0019252	BP-starch biosynthetic process	19	2	17	75	0.253333333	6.27471E-07	0.000229793
11	GO:0009311	BP-oligosaccharide metabolic process	21	4	17	188	0.111702128	6.54654E-07	0.000229793
12	GO:0006790	BP-sulfur compound metabolic process	36	13	23	380	0.094736842	6.75576E-07	0.000229793
13	GO:0005984	BP-disaccharide metabolic process	20	4	16	159	0.125786164	7.86906E-07	0.000229793
14	GO:0010103	BP-stomatal complex morphogenesis	13	0	13	65	0.2	8.3494E-07	0.000229793
15	GO:1901659	BP-glycosyl compound biosynthetic process	12	3	9	65	0.184615385	8.3494E-07	0.000229793
16	GO:0016556	BP-mRNA modification	14	1	13	77	0.181818182	8.5885E-07	0.000229793
17	GO:0044282	BP-small molecule catabolic process	29	21	8	224	0.129464286	8.76819E-07	0.000229793
18	GO:0009657	BP-plastid organization	30	3	27	176	0.170454545	9.13159E-07	0.000229793
19	GO:0005982	BP-starch metabolic process	20	2	18	90	0.222222222	9.22704E-07	0.000229793
20	GO:0006073	BP-cellular glucan metabolic process	32	6	26	349	0.091690544	9.73952E-07	0.000229793
**No**.	**KEGG pathway Id**	**KEGG pathway term**	**Sample gene number**			**Background gene number**	**Rich factor**	***P*****-value**	***P*****-adjust**
			**Total**	**Up**	**Down**				
1	GO:0009658	Porphyrin and chlorophyll metabolism	13	3	10	48	0.270833333	2.41796E-08	2.92573E-06
2	GO:0016054	Glyoxylate and dicarboxylate metabolism	16	5	11	112	0.142857143	7.18889E-06	0.000434928
3	GO:0046395	Photosynthesis	18	1	17	150	0.12	2.3227E-05	0.00093682

KEGG pathway enrichment analysis of the 1546 differentially expressed SAGs indicated that there were 633 SAGs associated with 121 KEGG pathways, with 3 pathways showing significant expression differences throughout this process (*P*-adjust < 0.05). The most significantly enriched pathways were “Porphyrin and chlorophyll metabolism” (13 genes), “Glyoxylate and dicarboxylate metabolism” (16 genes), and “Photosynthesis” (18 genes). Throughout the salt-stress period, the involved metabolic processes were mostly associated with nutrient recycling and photosynthesis, which correspond with the progression of the leaf senescence process (Table 1).

In addition, we also performed separate GO enrichment and KEGG pathway enrichment analyses for differentially expressed SAGs shared by salt- and dark-induced groups at three time points (3,001, 3,787, and 4,419 SAGs on day 2, 4, and 6, respectively), to assess these genes in more detail (Supplementary Figures 4, 5). The analysis showed that the changes in photosynthesis and nutrient recycling began to occur on day 2 of senescence in salt-stressed leaves. The metabolism of secondary substances was enhanced, with increased expression of many antioxidants and antioxidant enzymes involved in the senescence process associated with changes in plant hormone signal transduction. The genes involved in photosynthesis, light reactions, and carbon fixation in non-senesced leaves were down-regulated as leaves senesced, along with an enhancement of genes associated with amino acid metabolism, lipid metabolism, nutrient recycling, and secondary material metabolism over time.

### Dynamic SAG Expression During Leaf Senescence

**Figure 5:**
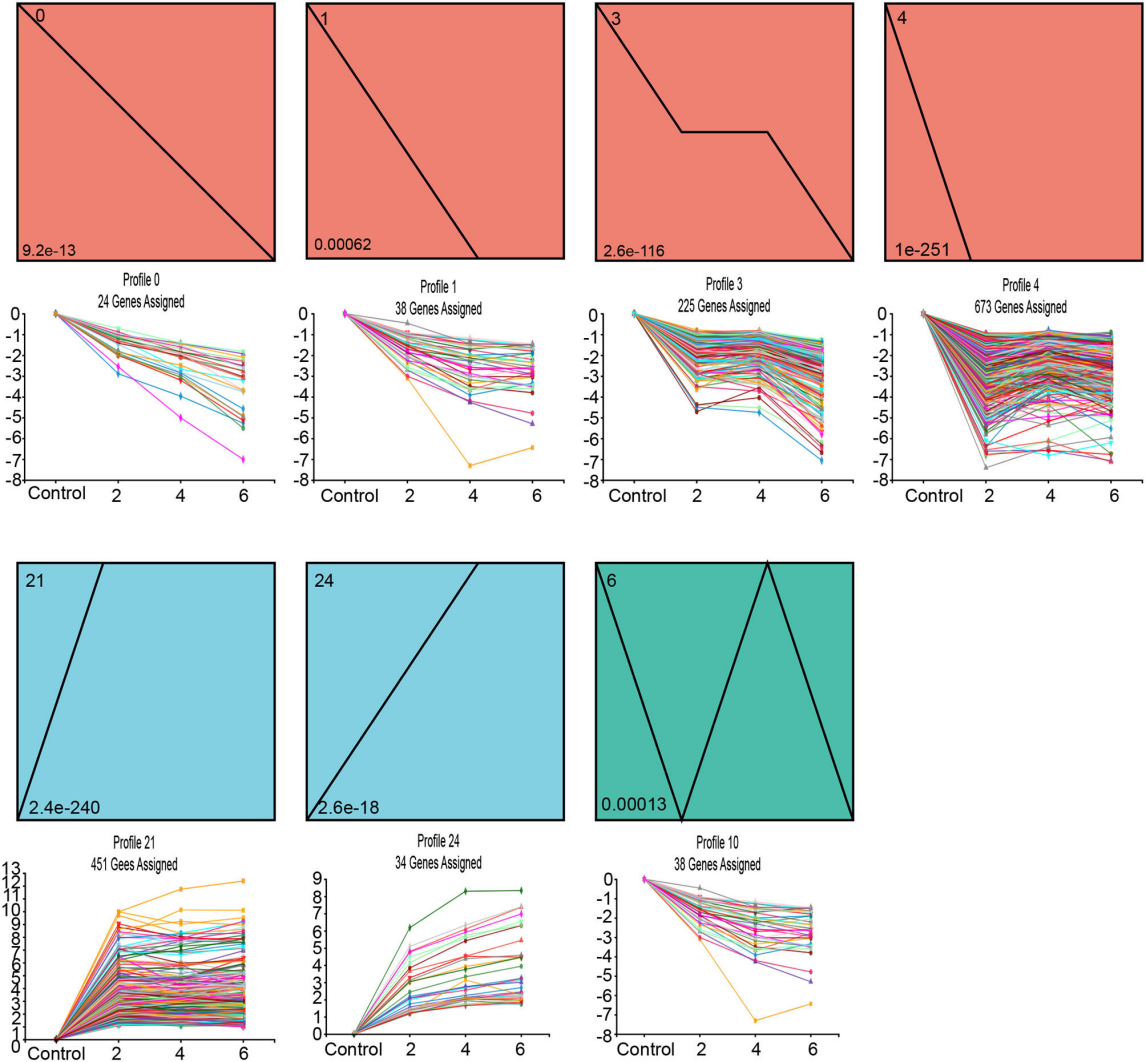
Short Time-series Expression Miner (STEM) analysis of darkness and salt treatment-enriched 1546 SAGs across three time points. Each square box indicates a type of expression profile, with the profile order on the upper left and the *P*-value on the bottom left. Only the significantly enriched cluster profile with a *P-adjust* < 0.05 threshold is shown.

In order to analyze the dynamic expression of 1546 SAGs at different time points (day 0, 2, 4, and 6) during leaf senescence, we used Short Time-series Expression Miner (STEM) for temporal pattern analysis. There were three main gene expression patterns observed (*P* < 0.05), including two up-regulated patterns (blue, profiles 21 and 24), four down-regulated patterns (orange-red, profiles 0, 1, 3, and 4), and one other pattern (green profiles 6) (Figure 5). Each profile contains genes with the same expression trend.

The KEGG pathway enrichment analysis of these three clusters revealed that the metabolic pathways of these genes differed among clusters. The genes from the up-regulated patterns of profiles 21 and 24 were associated with the organic substance catabolic process and amino acid metabolism, including cysteine & methionine metabolism and arginine & proline metabolism; in contrast, SAGs with down-regulated patterns corresponding to profiles 0, 1, 3, and 4 were enriched mainly in energy metabolism and carbohydrate metabolism (Supplementary Figure 6).

### Cluster Analysis

**Figure 6:**
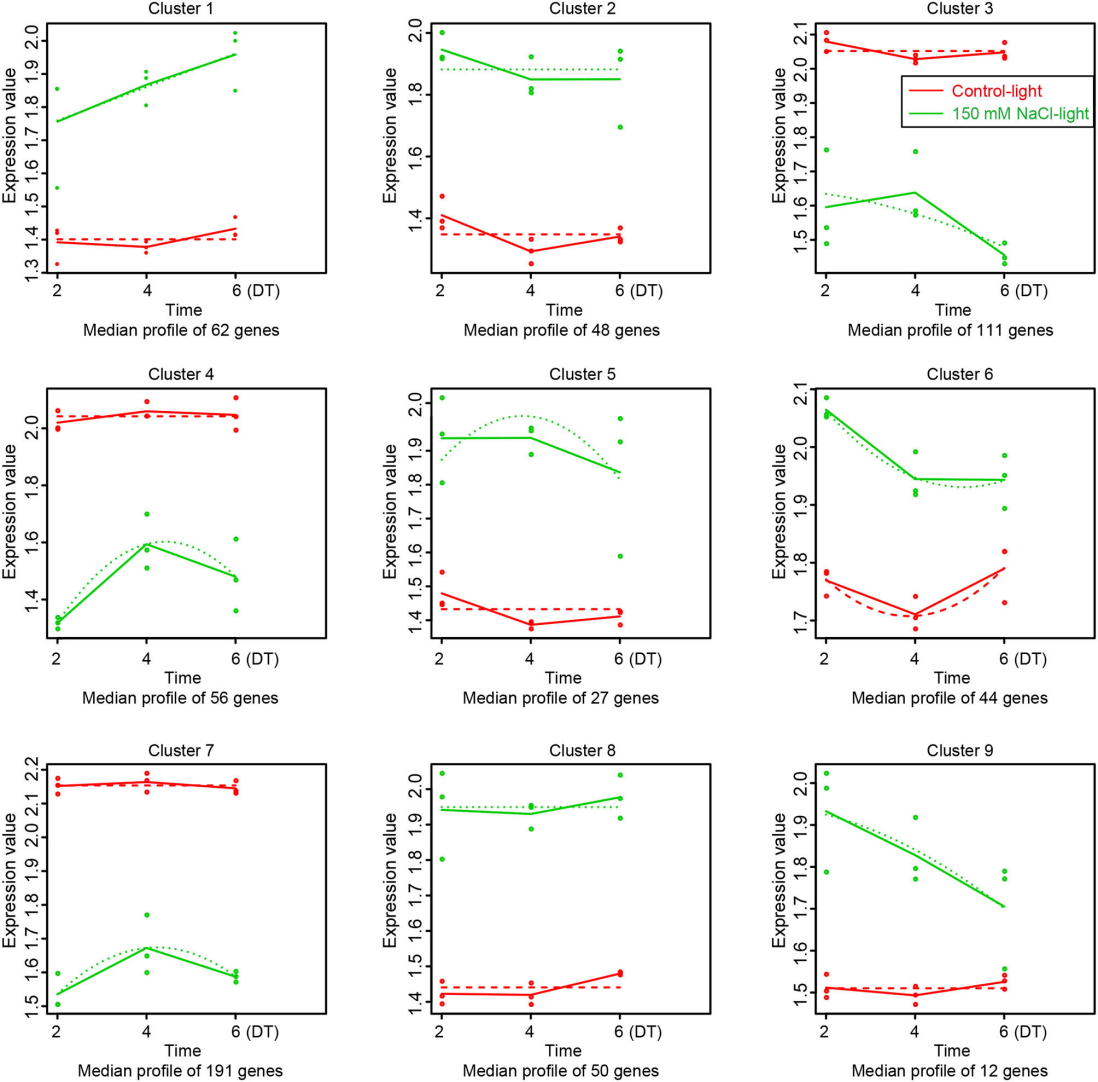
Data visualization with maSigPro (https://www.ncbi.nlm.nih.gov/pmc/articles/PMC4155246/). Each line chart shows the average expression profile of the gene clusters. Horizontal axis represents different time points, vertical axis represents average expression values. Different colored lines represent different groups (red and green lines represent the control-light and 150 mM NaCl-light groups, respectively). Dotted lines represent Fitted curves of the gene expression at each time point.

Based on MaSigPro (Version 1.56.0) analysis, we obtained the transcriptome profile of salt stress-induced leaf senescence for 2, 4, and 6 days in *Medicago truncatula*, and the results revealed 601 (9 clusters) SAGs which from 1546 SAGs (Figure 4D). The actual gene expression differences between the control-light groups and 150 mM NaCl-light are showed in Figure 6. Compared to the control-light group, the SAGs expression in each cluster of the 150 mM NaCl-light group varied within a specific pattern. These up-regulated genes were classified into six groups (clusters 1, 2, 5, 6, 8, and 9), while the down-regulated genes were classified into three groups (clusters 3, 4, and 7). It is noteworthy that 358 genes (59.6%) were enriched into these three clusters. Clustering of expression patterns of genes in all samples is shown in Supplementary Figure 7.

### Transcription Factor Analysis

**Figure 7:**
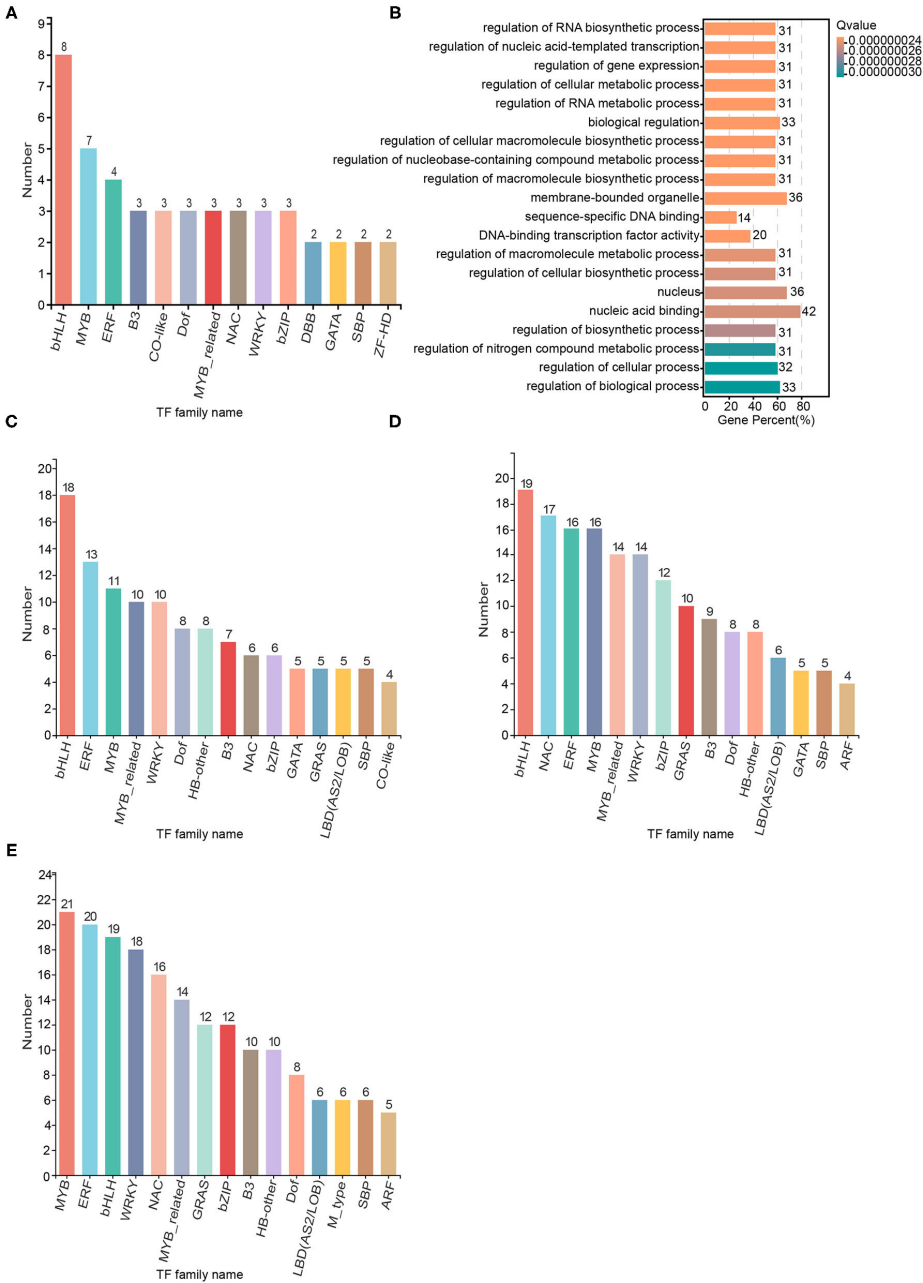
Transcription factor families of the senescence-associated genes (SAGs) on day 2, 4, and 6 shared by the darkness and salt treatments. Significantly enriched transcription factor families were identified by Hmmscan *E*-value (1.0E-5) and Blast *E*-value (1.0E-5). **(A)** The transcription factor families from 1546 darkness and salt treatment SAGs across three time points. **(B)** Scatter diagram of transcription factor families in **(A)** based on the Gene Ontology enrichment (GO) enrichment analysis. **(C)** Transcription factor families in the 3,001 darkness and salt treatment SAGs on day 2. **(D)** Transcription factor families in the 3,787 darkness and salt treatment SAGs on day 4. **(E)** Transcription factor families in the 4,419 darkness and salt treatment SAGs on day 6.

We used Plant TFDB 4.0 to perform TF prediction and statistical analysis of the 1546 SAGs identified. There were 146 TFs among the 1546 SAGs annotated by Plant TFDB 4.0, and the top three representative transcription families with the most members were bHLH (eight members), MYB (seven members), and ERF (seven members). The transcription factor families NAC, B3, CO-like, Dof, MYB-related, WRKY, and bZIP each had three members included (Figure 7A). Most of these TFs were down-regulated throughout the senescence process, but the NAC TF family was up-regulated (Supplementary Table 13). GO enrichment analysis revealed that the TFs were involved in biological regulation, organelle, cell part and transcription regulator activity categories (Figure 7B). Furthermore, we identified 351, 440, and 545 TFs among the significantly differentially expressed SAGs shared by darkness and salt treatment on day 2, 4, and 6, respectively (Figures 7C–E, Supplementary Table 7). The most differentially expressed senescence-associated TFs belonged to the bHLH, ERF, MYB, NAC, and WRKY TF families, which are the major families known to be involved in the regulation of senescence.

### Gene Function Verification by *Agrobacterium*-Mediated Transient Expression

To identify the functions of differentially expressed SAGs in our results, we performed a transient overexpression assay in *Nicotiana benthamiana*. Based on the expression patterns of the 146 TFs among the different clusters (Figure 5), eight senescence-associated TFs were chosen, and their constructs were then agro-infiltrated into *Nicotiana benthamiana* leaves (Table 2).

**Table 2:** Screening of TFs for gene function identification.

**Number**	***Medicago truncatula* gene Id**	***Arabidopsis thaliana* gene stable Id**	***Arabidopsis thaliana* gene name**	**Identity %**
1	MTR_5g011120	AT4G22920	*SGR1* (Positive Control)	61.9
2	MTR_3g088795	AT1G44000	*SGRL*	48.5
3	MTR_2g023930	AT1G62300	*WRKY6*	50.1
4	MTR_1g090723	AT3G10500	*NTL4*	29.3
5	MTR_4g081870	AT1G69490	*NAP*	59.3
6	MTR_4g111975	AT1G49010	*At MYBL*	57.6
7	MTR_6g012670	AT5G39610	*ANAC092*	49.5
8	MTR_3g088110	AT1G01720	*NAC002*	73.0
9	MTR_2g079710	AT4G27440	*PORB*	82.3

**Figure 8:**
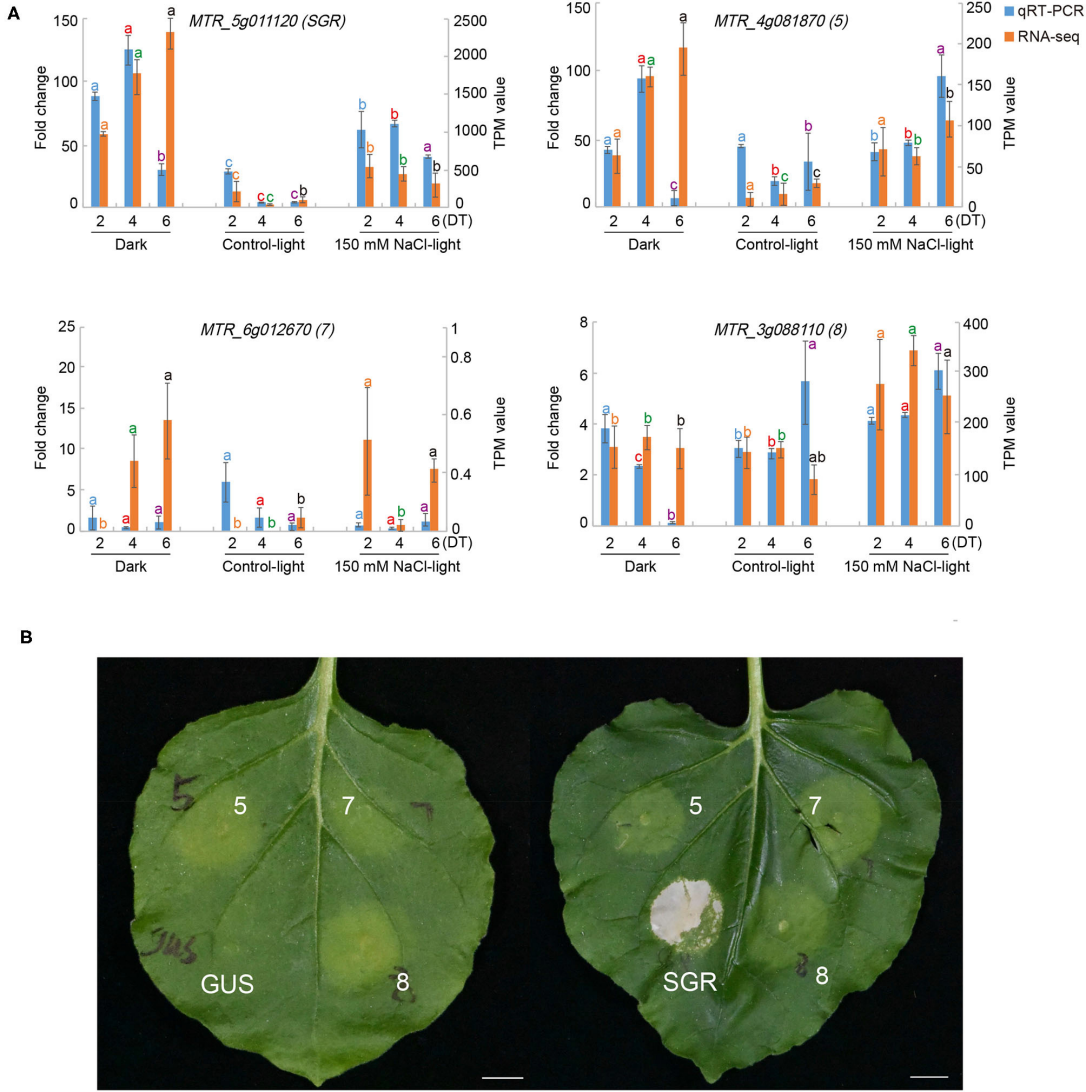
Functional validation of selected senescence-associated genes (SAGs) using an *Agrobacterium*-mediated transient expression assay. **(A)** The relative expression fold change of selected SAGs under dark (Dark), normal light (Control-light), and 150 mM NaCl (150 mM NaCl-light) conditions on day 2, 4, and 6 compared with day 0 by RNA-seq and qRT-PCR. **(B)** Representative *Nicotiana benthamiana* leaf senescence symptoms 24 h after infiltration with different constructs encoding MTR_5g011120 (*SGR*), MTR_4g081870 (5), MTR_6g012670 (7), MTR_3g088110 (8), and empty vector with GUS, respectively.

We found that the green leaf spots infiltrated by the number 5, 7, 8, and SGR constructs (MTR_4g081870, MTR_6g012670, MTR_3g088110, and MTR_5g011120, respectively), began to turn yellow 24 h after being injected with *Agrobacterium tumefaciens* (Figure 8), but no change occurred in the leaves injected with H_2_O or blank vectors. These results indicated that the three TFs may play important roles in the leaf senescence process.

We predicted that the TF MTR_3g088110 co-regulates 56, 69, and 94 target genes on day 2, 4, and 6, respectively (Supplementary Figure 8). Moreover, these genes were associated with the response to oxygen-containing compounds, carbohydrate biosynthetic process, vacuole organization, sphingolipid metabolism, and plant hormone signal transduction.

### Data Integration Analysis

To gain a comprehensive understanding of the leaf senescence process, we integrated transcriptomic data with physiological and biochemical data for a combined analysis. We found that the expression levels of the differentially expressed SAGs were closely related to plant metabolite activity. Many genes related to lipid oxidation and antioxidant defense were differentially expressed, which is consistent with the increased H_2_O_2_ contents observed. We also found differentially expressed SAGs involved in plant hormone signal transduction along with an increase in ABA content at the start of the stress treatment and then a decrease as leaf senescence proceeded (Supplementary Figure 9A, Supplementary Table 14). In addition, during leaf senescence, the genes related to nutrient cycling were abundantly expressed, indicating the nutrients' movement and redistribution. To elucidate the relationship between leaf senescence and leaf nutrients, we focused on the expression of SAGs related to protein and amino acid metabolism. We found 247 protein-metabolism-related genes among the 1546 SAGs, out of which 135 were related to amino acid metabolism, and most of them were down-regulated. We performed a STEM analysis of these genes and found that compared with the control-light group, most of them in the salt-induced group were down-regulated across the three time points, suggesting protein metabolism increased as leaves senesced (Supplementary Figure 9B).

## Discussion

In this study, we examined salt stress-induced senescence in *Medicago truncatula* leaves using a time-course analysis of the expression patterns of SAGs. To obtain accurate SAG expression data during the complex process of senescence, we sampled highly controlled repeatable detached leaves and measured them at many time points. As a positive control for our data analysis, we also included a dark-induced senescence treatment. This experimental design enabled the superimposition of the data from both treatments and helped us to obtain time-course differential expression network data for those genes specifically associated with salt-induced leaf senescence.

Our data showed a significant downward trend in chlorophyll content during leaf senescence from 0 to 6 days under salt stress, which corresponds to the observed leaf senescence symptoms. We also found that 252 SAGs are related to chlorophyll and chloroplast metabolism (Supplementary Figure 9). Over a long period of time, many genes encoding proteins involved in the chlorophyll catabolism pathway have been discovered and cloned, including *STAY-GREEN* (Zhou et al., 2011a), *SAG12* (Noh and Amasino, 1999), and *WRKY53* (Zentgraf et al., 2010). Similarly, many enzymes related to chlorophyll degradation have been identified, such as chlorophyllase (AtCLH), pheophorbide a oxygenase (AtPaO), and red chlorophyll catabolite reductase (RCCR) (Mach et al., 2001; Benedetti and Arruda, 2002; PruŽinská et al., 2003).

H_2_O_2_ is commonly used as an indicator of salt stress in plants; thus, we examined the levels of this substances in our assay (Figure 2B). In our experiment, the H_2_O_2_ content accumulated throughout leaf senescence, although with some variation. H_2_O_2_ is a hub for ROS interconversion, which can directly or indirectly oxidize biomolecules, such as intracellular nucleic acids and proteins, and damage cell membranes, thereby accelerating cellular senescence and disintegration (Bieker et al., 2012; Chen et al., 2012). The structural integrity and physiological functions of the cell membrane were disrupted when the leaf cells were subjected to oxidative damage (Halliwell, 1984).

Leaf senescence is associated with many kinds of plant hormones, and ABA is an important stress hormone involved in leaf senescence (Hung and Kao, 2004). We assessed ABA levels throughout the salt-induced leaf senescence process and found that ABA increased during leaf senescence (Figure 2C). ABA can promote elevated protein hydrolase and RNase activities and accelerate protein and nucleic acid degradation, thereby reducing photosynthetic rates and promoting leaf senescence (Marschner, 2011). Liang et al. (2014) identified OsNAP, an essential component of the ABA-mediated plant senescence signaling pathway in rice, and elucidated the molecular regulatory mechanism of ABA-mediated senescence in rice leaves for the first time (Liang et al., 2014). Similarly, Mao et al. (2017) revealed the regulatory network underlying ABA synthesis using the ABA-NAC-SAGs model, providing a new theoretical basis for the study of the molecular mechanisms of ABA regulation of leaf senescence (Mao et al., 2017).

During plant senescence, gene expression levels are closely related to metabolite content, with many metabolites involved in amino acid and lipid metabolism increasing and those involved in chloroplast metabolism decreasing (Breeze et al., 2011; Moschen et al., 2016; Sekhon et al., 2019). In our assay, we identified 3,001, 3,787, and 4,419 SAGs in total on day 2, 4, and 6, respectively, through transcriptomic analysis of salt-treated detached leaves, which appear to be involved in multiple signaling pathways and regulatory networks in leaf senescence. GO and KEGG enrichment analyses indicated that differentially expressed genes were mainly enriched in lipid or carbohydrate metabolism, amino acid metabolism and synthesis of secondary metabolites. In addition, genes involved in nutrient recycling and photosynthesis pathways were significantly enriched. Changes in the expression of these genes may impact the final forage quality in agricultural production.

TFs have been reported to play central roles in leaf senescence (Balazadeh et al., 2008). In recent years, many senescence-associated TFs have been discovered in model plants. For example, Guo et al. (2004) identified at least 96 TFs with 3-fold changes in gene expression during leaf senescence (Guo et al., 2004). In our study, we predicted TFs from among significantly differentially expressed SAGs and found that many of these predicted TFs belonged to the bHLH, MYB, ERF, and NAC families, which are the major families known to be involved in regulating senescence. We selected nine TFs with significant expression differences, including members of the NAC, MYB, WRKY, and NAP families, and transiently expressed them in *Nicotiana benthamiana* to verify their functions. Three of the selected TFs caused senescence phenotypes in *Nicotiana benthamiana* leaves: MTR_4g081870, MTR_6g012670, MTR_3g088110. We found the high sequence identities of MTR_4g081870, MTR_6g012670, and MTR_3g088110 from *Medicago truncatula* with AT1G69490 (NAP), AT5G39610 (ANAC092), and AT1G01720 (NAC002) from *Arabidopsis thaliana*, respectively. Therefore, MTR_4g081870, MTR_6g012670, and MTR_3g088110 may be involved in the regulation of leaf senescence. Further bioinformatic analysis and laboratory experiments may help to characterize their functions in more details.

Members of the NAC TF family in *Arabidopsis thaliana* play an important role in natural senescence or the biotic and abiotic stress-induced leaf senescence process. NAC1 is regulated by miR164, which regulates leaf senescence and causes cell death. However, *Arabidopsis thaliana* mutants that lack NAC1 did not show significant leaf senescence, and thus, NAC1 may not play a key role in leaf senescence (Guo et al., 2005; D'haeseleer et al., 2011). AT1G69490 (AtNAP) has been confirmed to be directly related to leaf senescence (Guo and Gan, 2006). AT5G39610 (ANAC092) plays a major role in the leaf senescence-related gene regulatory network and is an important network element that regulates salt stress and promotes senescence (Balazadeh et al., 2010). Garapati et al. (2015) suggested that AT1G01720 (NAC002) executes its physiological role by affecting both key chloroplast maintenance and senescence-promoting TFs (Garapati et al., 2015). Therefore, NAC TF family may also play a critical role in salt induced leaf senescence in *Medicago truncatula*, although the details of molecular mechanism is not well-known.

We also found that MTR_2g091190 (TF bHLH30), MTR_1g052470 (TF bHLH122-like protein), and MTR_1g080890 (TF UNE12) were highly up-regulated during leaf senescence, but these genes have not been reported to be directly related to plant senescence until now. MTR_3g103580, which is a member of the MYB family, showed high expression levels during leaf senescence in *Medicago truncatula*. This TF has high sequence similarity to AT1G10070 (BCAT2) from *Arabidopsis thaliana*. BCAT2 encodes a chloroplast branched-chain amino acid aminotransferase and is involved in cell wall development (Angelovici et al., 2013). Therefore, we have reason to believe that this TF of MTR_3g103580 regulates the metabolic activity of chloroplasts.

Many studies have shown that ethylene is involved in leaf abscission and may thus be associated with the initiation of leaf senescence (Aharoni and Lieberman, 1979). We also identified one ERF TF that showed high expression levels. MTR_1g074370 (ethylene-responsive TF 1B) had high sequence similarity to AT3G23240 (ERF1B) from *Arabidopsis thaliana*. ERF1 regulates ethylene overproduction and is involved in the ethylene signaling cascade. ERF1 was highly induced by high salinity stress in *Arabidopsis* and plays a positive role in salt stress tolerance (Lorenzo et al., 2003; Cheng et al., 2013, 2017). More laboratory experiments may help us understand their functions in more details.

Plant senescence is an important part of plant growth and development, and the timing of crop senescence profoundly affects the stability of crop yield, quality, and regional distribution. Breeders consider appropriate senescence time to be a key agronomic trait and thus aim to obtain superior germplasms with ideal senescence times in order to improve agricultural production. Alfalfa (*Medicago sativa L*.) is one of the world's most important forage crops owing to its high yield, high nutritional quality and wide adaptability. Additionally, most of the nutrients in alfalfa are stored in its leaves, and leaf senescence may therefore greatly affect the overall nutritional quality of alfalfa, especially when induced by external factors, such as salinity, drought, or cold stress, which can lead to premature aging of leaves. Therefore, preventing premature aging or senescence to increase biomass accumulation is profoundly important for improving alfalfa production. Furthermore, our previous research has shown that silencing the endogenous *SGR* gene (*MsSGR*) in alfalfa can result in the retention of more than 50% of their chlorophyll content during senescence, thus significantly increasing the crude protein contents compared to control lines (Zhou et al., 2011a).

In summary, *M. truncatula*, a close relative of alfalfa (*M. sativa*), has been widely used as a model in legume biology and is therefore a valuable experimental system for studying forage senescence. We have presented and analyzed a highly informative gene expression dataset over the course of senescence under salt stress and darkness. Our knowledge about the mechanisms regulating leaf senescence can be applied to improving the biomass and quality of forage under salt stress through future forage breeding.

## Data Availability Statement

The original contributions presented in the study are publicly available. This data can be found at: NCBI repository, accession number: PRJNA701852 (https://www.ncbi.nlm.nih.gov/bioproject/701852).

## Author Contributions

Z-YW, MC, and SD designed and planned the experiments. SD and LS performed the experiments. SD analyzed the data and wrote the manuscript. MC, HX, and Z-YW revised the manuscript. All authors read, revised, and approved the final manuscript.

## Conflict of Interest

The authors declare that the research was conducted in the absence of any commercial or financial relationships that could be construed as a potential conflict of interest.

## Supplementary Material

Supplementary Figure 1 The sampled leaf tissues and the relationship between leaf senescence and different NaCl concentration treatments. **(A)** The leaves detached for treatment-induced senescence. **(B)** The relationship between leaf senescence and different concentrations of NaCl.

Supplementary Figure 2 The validation of RNA-seq data via qRT-PCR. The relative expression fold change of the representative senescence-associated genes (SAGs) from different clusters in Figure 5 in different conditions compared with day 0.

Supplementary Figure 3 Significant Gene Ontology (GO) distribution for molecular function of 1546 senescence-associated genes (SAGs) in Figure 4D.

Supplementary Figure 4 Gene Ontology (GO) enrichment analysis of senescence-associated genes (SAGs) shared by darkness and salt-induced leaf senescence on day 2 (**A** for Figure 4A, 3011 enrichment analysis of genes enrichment analysis), 4 (**B** for Figure 4B, 3787 enrichment analysis of genes enrichment analysis), and 6 (**C** for Figure 4C, enrichment analysis of 4419 genes) treatment in detached *Medicago truncatula* leaves. Only the GO term corresponding with the top 20 enrichment levels are shown. GO term are shown along the vertical axis. In contrast, the horizontal axis represents the enrichment factor, i.e., the ratio of the number of genes/transcripts (sample number) enriched in this pathway relative to the number of annotated genes/transcripts (background number). The greater the enrichment factor, the greater the degree of enrichment. The size of the point indicates the number of genes in this pathway, while the colors of the points correspond to different *P*-value ranges.

Supplementary Figure 5 Scatter diagram based on the Kyoto Encyclopedia of Genes and Genomes (KEGG) pathway enrichment analysis of the senescence-associated genes (SAGs) shared by darkness and salt-induced leaf senescence on day 2 (**A** for 3,011 genes), 4 (**B** for 3,787 genes), and 6 (**C** for 4,419 genes) in detached *Medicago truncatula* leaves. Only the KEGG pathways within the top 20 enrichment levels are displayed. Pathway names are shown along the vertical axis, while the horizontal axis represents the enrichment factor, i.e., the ratio of the number of genes/transcripts (sample number) enriched in this pathway relative to the number of annotated genes/transcripts (background number). The greater the enrichment factor, the greater the degree of enrichment. The size of the point indicates the number of genes in this pathway, while the colors of the points correspond to different *P-adjust* value ranges.

Supplementary Figure 6 Scatter diagram based on the Kyoto Encyclopedia of Genes and Genomes (KEGG) pathway enrichment analysis of the senescence-associated genes (SAGs) in Figure 5 for up-regulated **(A)** and down-regulated genes **(B)**.

Supplementary Figure 7 The maSigPro analysis of Differentially expressed senescence-associated genes (SAGs) in all samples. The horizontal axis represents specific samples, the vertical axis represents the expression values after treatment log10(x+1), and the different colored lines represent the trend of different genes in all samples.

Supplementary Figure 8 A hypothetical network of transcription factor MTR_3g088110. It showed the predicted potential target genes of transcription factors MTR_3g088110 across three time points: MTR_3g088110-2, MTR_3g088110-4, and MTR_3g088110-6 display the genes from day 2 Figure 4A 3011genes, day 4 Figure 4B 3787genes, and day 6 Figure 4C 4419genes co-regulated with MTR_3g088110, respectively.

Supplementary Figure 9 Differentially expressed senescence-associated genes (SAGs) related to physiological and biochemical indicators. **(A)** The number of genes from darkness and salt treatment enriched 1546 SAGs was associated with physiological and biochemical changes during salt induced leaf senescence. **(B)** Short Time-series Expression Miner (STEM) analysis of the genes in **(A)**.
