# Supplementary figures and images for: Comparative Transcriptome Analysis of Salt Stress-Induced Leaf Senescence in Medicago truncatula
Source: Front Plant Sci. 2021 Jul 9;12:666660. doi: 10.3389/fpls.2021.666660 (PMC8299074; doi:10.3389/fpls.2021.666660)

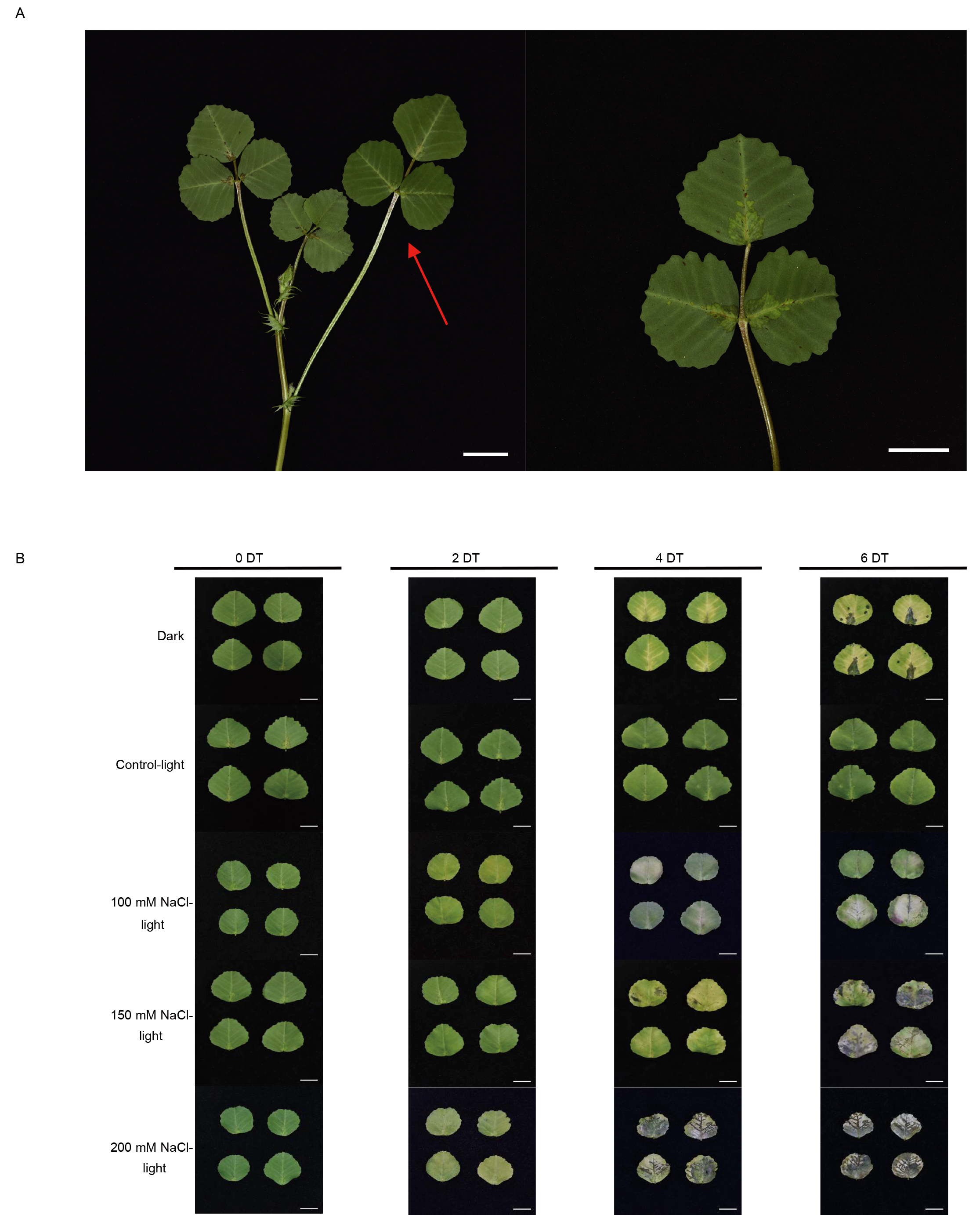

Supplement: Supplementary Figure 1 — The sampled leaf tissues and the relationship between leaf senescence and different NaCl concentration treatments. (A) The leaves detached for treatment-induced senescence. (B) The relationship between leaf senescence and different concentrations of NaCl. [file Data_Sheet_1.zip › supplementary files/Supplementary Figure 1.tif]

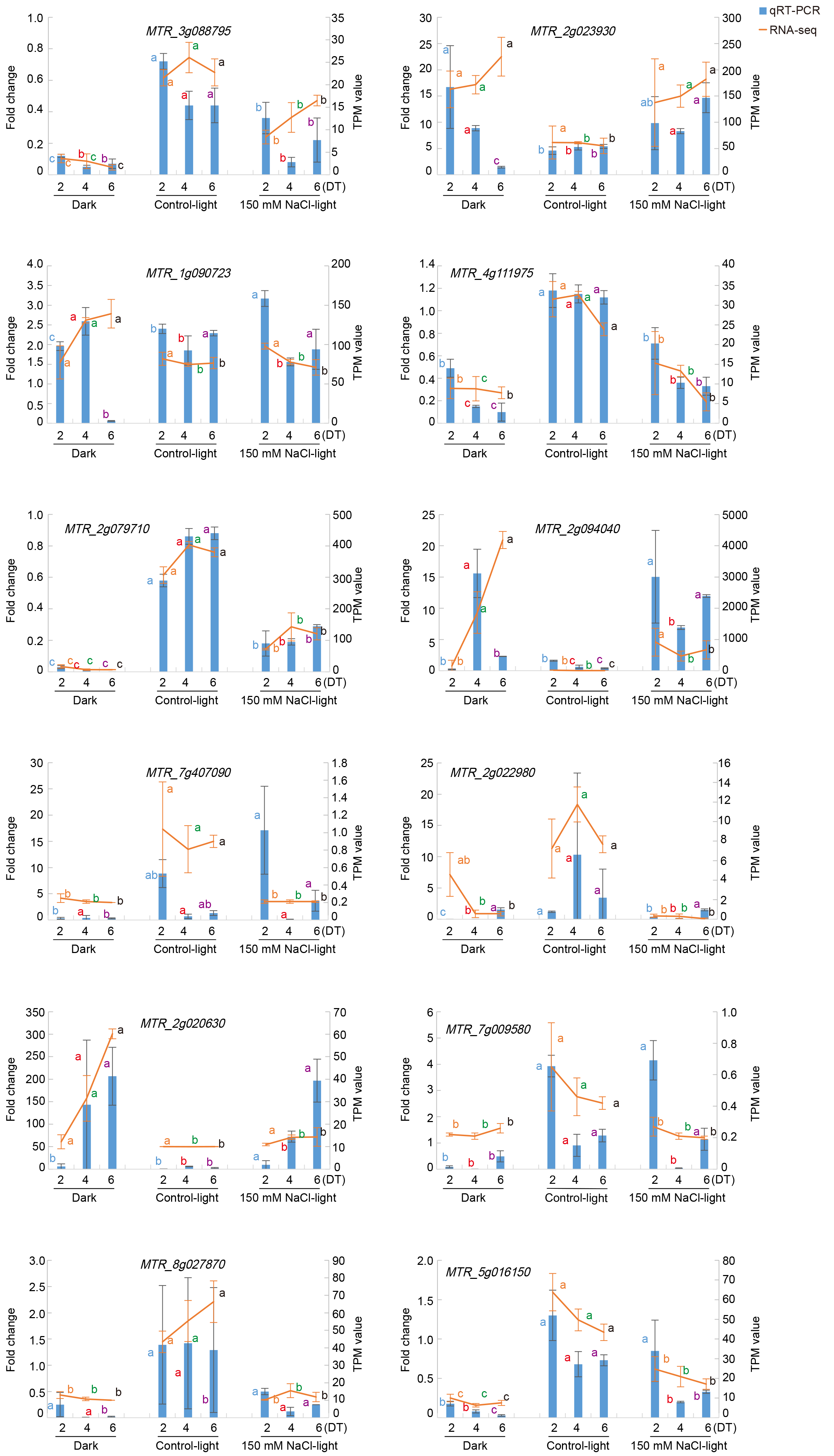

Supplement: Supplementary Figure 1 — The sampled leaf tissues and the relationship between leaf senescence and different NaCl concentration treatments. (A) The leaves detached for treatment-induced senescence. (B) The relationship between leaf senescence and different concentrations of NaCl. [file Data_Sheet_1.zip › supplementary files/Supplementary Figure 2.tif]

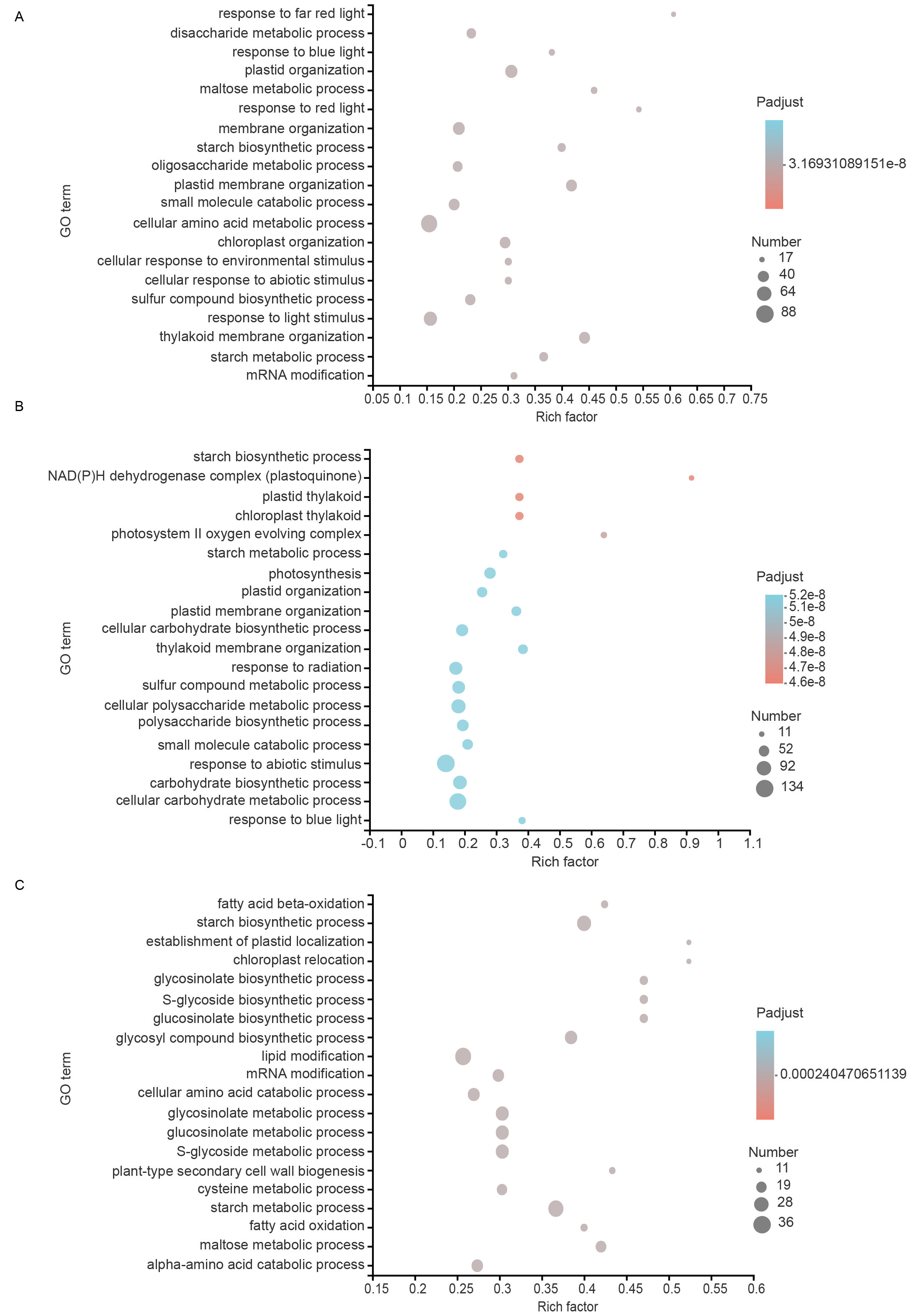

Supplement: Supplementary Figure 1 — The sampled leaf tissues and the relationship between leaf senescence and different NaCl concentration treatments. (A) The leaves detached for treatment-induced senescence. (B) The relationship between leaf senescence and different concentrations of NaCl. [file Data_Sheet_1.zip › supplementary files/Supplementary Figure 4.tif]

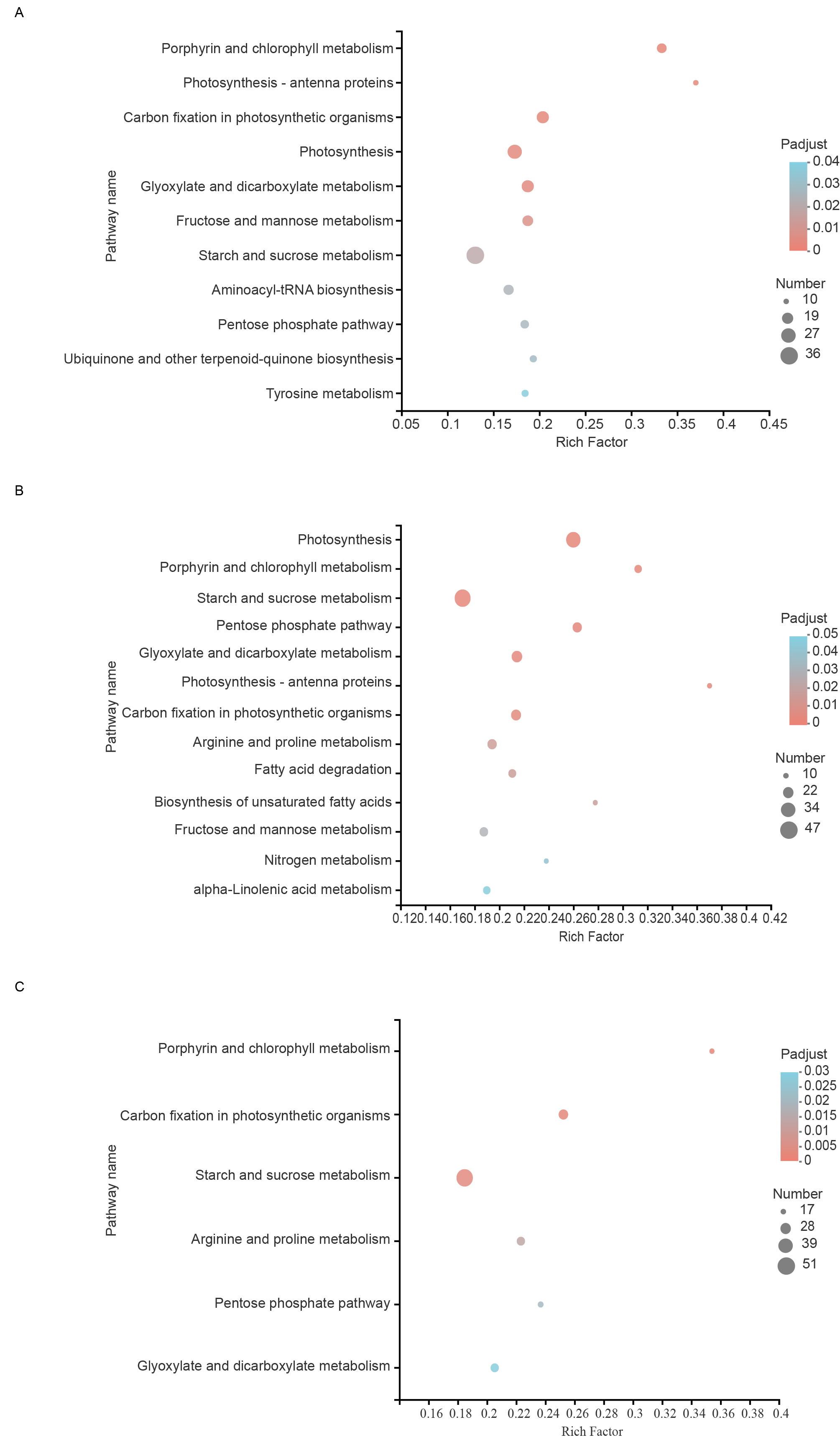

Supplement: Supplementary Figure 1 — The sampled leaf tissues and the relationship between leaf senescence and different NaCl concentration treatments. (A) The leaves detached for treatment-induced senescence. (B) The relationship between leaf senescence and different concentrations of NaCl. [file Data_Sheet_1.zip › supplementary files/Supplementary Figure 5.tif]

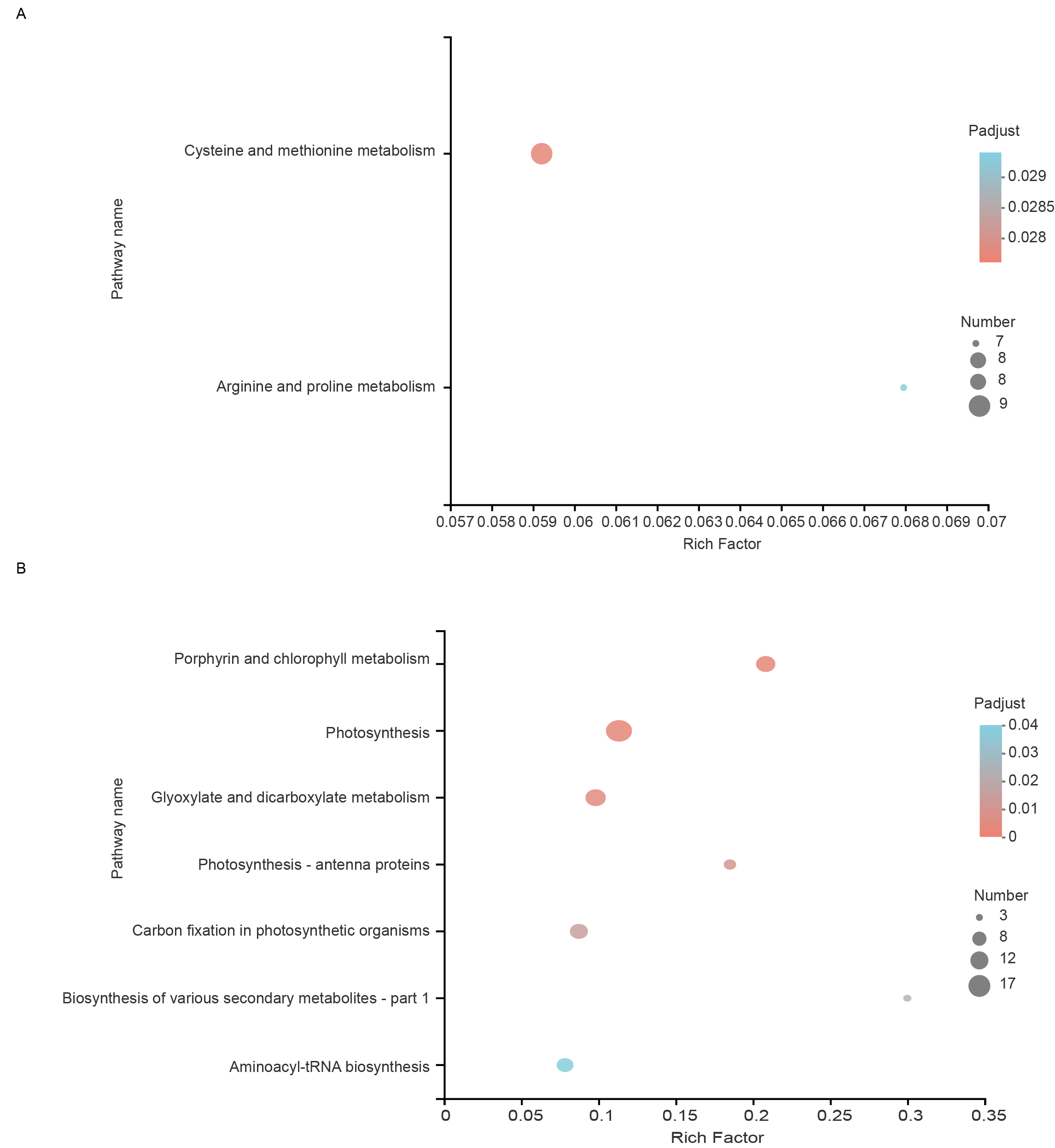

Supplement: Supplementary Figure 1 — The sampled leaf tissues and the relationship between leaf senescence and different NaCl concentration treatments. (A) The leaves detached for treatment-induced senescence. (B) The relationship between leaf senescence and different concentrations of NaCl. [file Data_Sheet_1.zip › supplementary files/Supplementary Figure 6.tif]

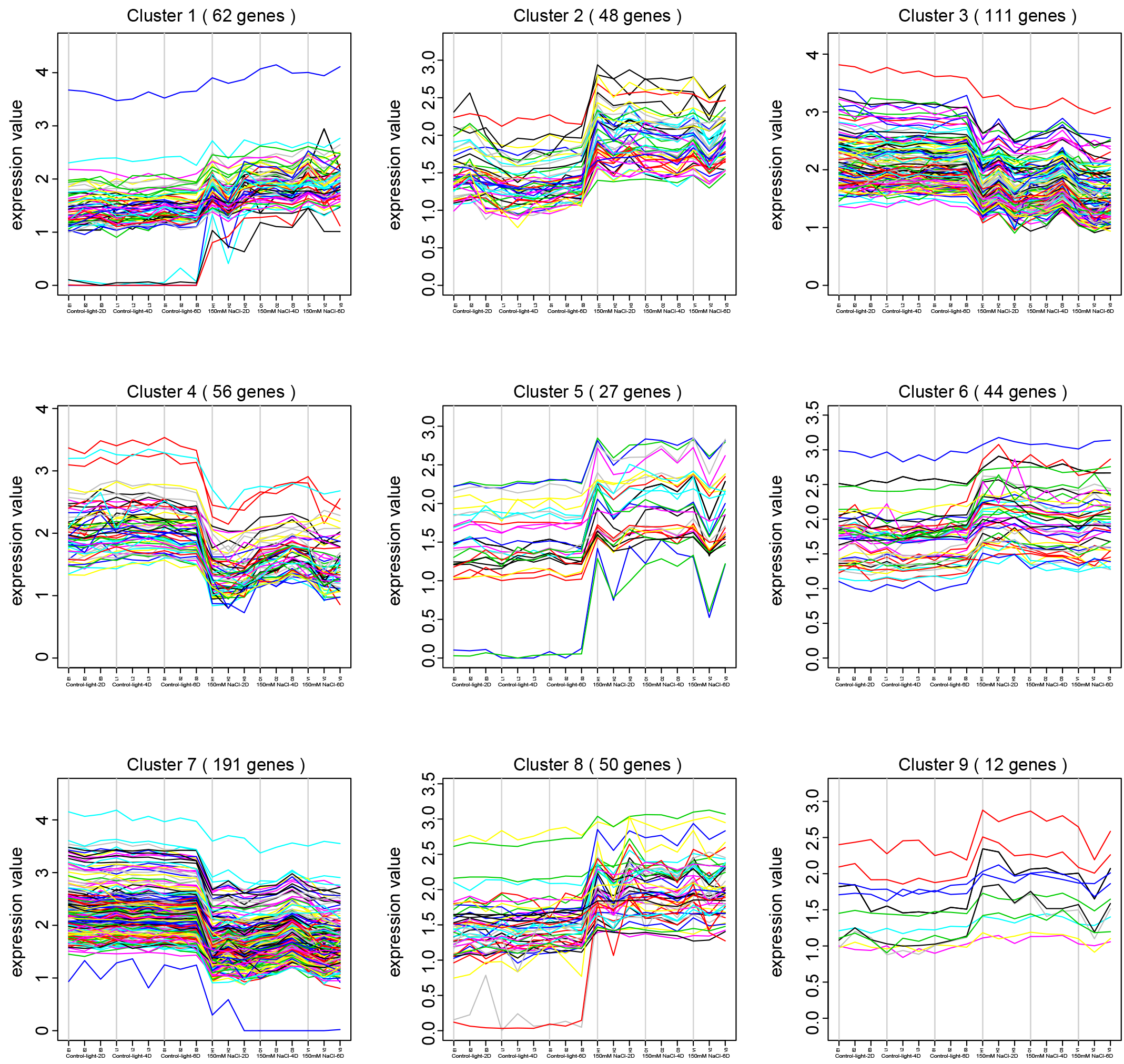

Supplement: Supplementary Figure 1 — The sampled leaf tissues and the relationship between leaf senescence and different NaCl concentration treatments. (A) The leaves detached for treatment-induced senescence. (B) The relationship between leaf senescence and different concentrations of NaCl. [file Data_Sheet_1.zip › supplementary files/Supplementary Figure 7.tif]

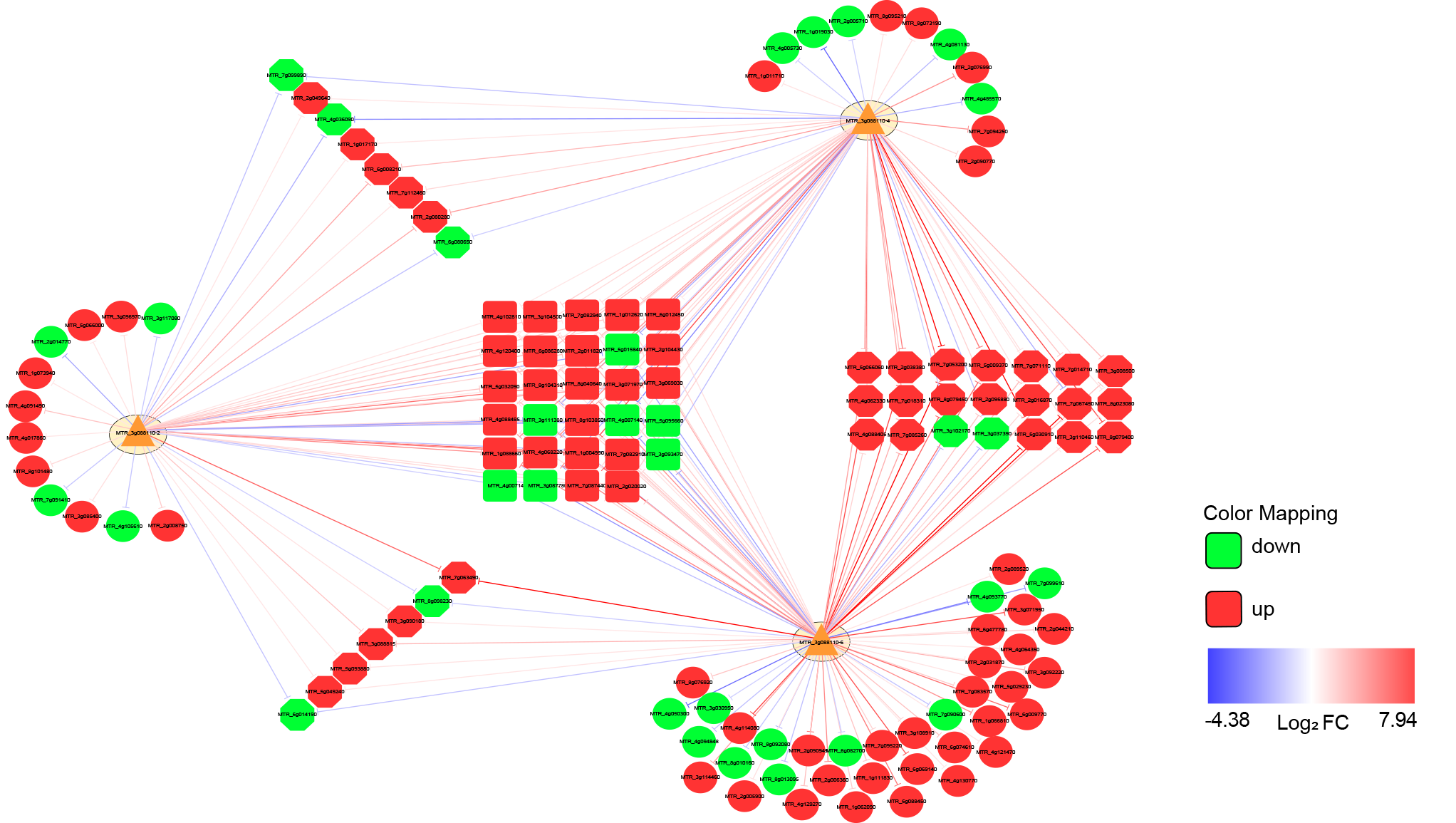

Supplement: Supplementary Figure 1 — The sampled leaf tissues and the relationship between leaf senescence and different NaCl concentration treatments. (A) The leaves detached for treatment-induced senescence. (B) The relationship between leaf senescence and different concentrations of NaCl. [file Data_Sheet_1.zip › supplementary files/Supplementary Figure 8.tif]

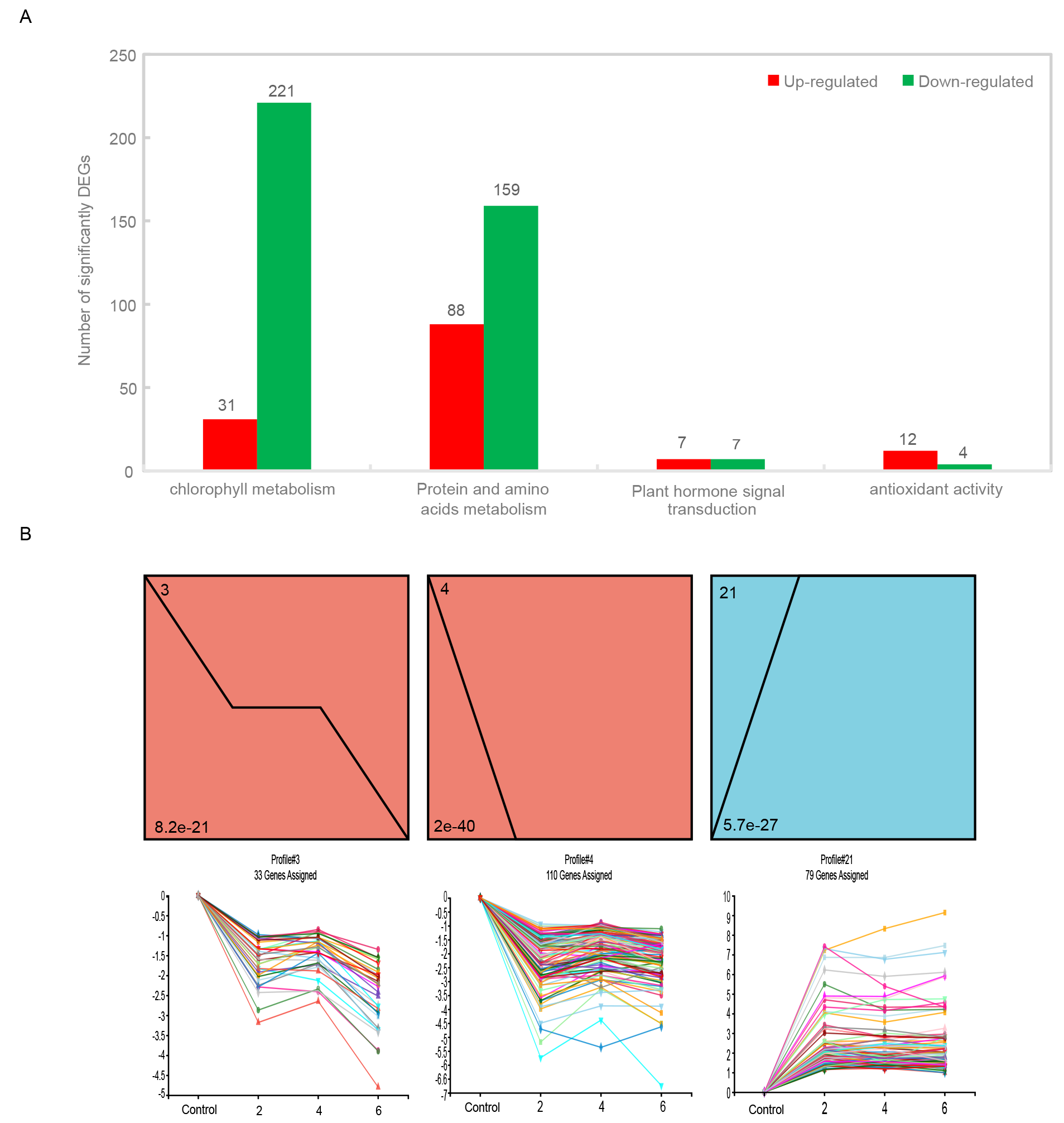

Supplement: Supplementary Figure 1 — The sampled leaf tissues and the relationship between leaf senescence and different NaCl concentration treatments. (A) The leaves detached for treatment-induced senescence. (B) The relationship between leaf senescence and different concentrations of NaCl. [file Data_Sheet_1.zip › supplementary files/Supplementary Figure 9.tif]
